# Parenting and personality disorder: An overview and meta-synthesis of systematic reviews

**DOI:** 10.1371/journal.pone.0223038

**Published:** 2019-10-01

**Authors:** Kayla R. Steele, Michelle L. Townsend, Brin F. S. Grenyer

**Affiliations:** School of Psychology, Illawarra Health and Medical Research Institute, University of Wollongong, Wollongong, New South Wales, Australia; King Fahd University of Petroleum & Minerals, SAUDI ARABIA

## Abstract

**Background/Objectives:**

Maladaptive parenting (including childhood maltreatment, abuse and neglect) has been implicated in the scientific literature exploring the aetiology of personality disorder, particularly borderline personality disorder (BPD). Our primary objective was to summarise the evidence on the relationship between parenting and personality disorder, assisting clinical decision-makers to translate this research into clinical policy and practice.

**Methods:**

We conducted an overview of systematic reviews that assessed individuals with personality disorder pathology for experiences of maladaptive parenting, compared to psychiatric or healthy comparisons/controls, and the impact on psychopathological and relational outcomes. Systematic literature searches were conducted in Scopus, Web of Science, MEDLINE, PsycINFO, and by hand in August 2018. Methodological quality was assessed using the CASP systematic review checklist, and results were qualitatively synthesised. A pre-determined protocol was registered in Prospective Register of Systematic Reviews (PROSPERO 2019:CRD42018096177).

**Results:**

Of the 312 identified records, 293 abstracts were screened, 36 full-text articles were retrieved and eight systematic reviews met pre-determined criteria for qualitative synthesises. The majority of studies reported outcomes related to BPD (*n* = 7), and study design, methodology and quality varied. Within the eight systematic reviews there were 211 primary studies, of which 140 (66.35%) met eligibility criteria for inclusion in this overview. Eligible primary studies reported on 121,895 adult, child/adolescent and parent-offspring participants, with most studies focused on borderline personality pathology (*n* = 100, 71.43%). Study design and methodology also varied for these studies. Overall, five systematic reviews overwhelming found that maladaptive parenting was a psychosocial risk factor for the development of borderline personality pathology, and three studies found that borderline personality pathology was associated with maladaptive parenting, and negative offspring and parenting-offspring outcomes.

**Conclusions:**

In light of these findings, we recommend greater emphasis on parenting in clinical practice and the development of parenting interventions for individuals with personality disorder. However, our understanding is limited by the heterogeneity and varying quality of the evidence, and as such, future research utilising more rigorous research methodology is needed.

## Introduction

Personality disorder is a complex mental illness that emerges in the context of relationships with the self and others, and is characterised by marked distress or impairment in response to a pervasive, inflexible and enduring pattern of inner experience and behaviour that deviates markedly from the expectations of the individual’s cultural norms [1]. Globally, personality disorder affects 6.1% of the general population [2] and as such, is considered a mental health priority area [3]. The most frequently reported personality disorder in primary care and mental health settings is Borderline Personality Disorder (BPD). Up to 23% of outpatients and 43% of inpatients in Australian mental health services meet criteria for BPD [4]. High rates of clinical presentation also partly explains inflated representation of BPD in the personality disorder and mental health literature more generally. Although BPD is thought to occur equally amongst men and women in the general population [5], women are disproportionately represented in clinical settings (comprising of up to 75% of those given a BPD diagnosis [6]). For individuals with BPD, emotional dysregulation, high levels of impulsivity leading to self-harm and suicidality, and disturbed interpersonal functioning, are thought to lead to difficulties in forming and maintain interpersonal and interpersonal relationships [1].

Although the exact aetiology of BPD remains unclear, contemporary models recognise that the disorder emerges from an interactive web of genetic, neural, behavioural, family and social pathways [7]. Many of these pathways converge within the parent-offspring relationship, and as such, this interaction is an important context for the pathogenesis of BPD. Family studies [8, 9] have found a 4 to 20-fold increase in BPD diagnosis and traits in first-degree relatives compared to the general population. Whilst twin studies report heritability estimates ranging from 42% for BPD features [10] to 69% for BPD diagnosis [11]. Consequently, it is hypothesised that children of parents with BPD may inherit genes predisposing them to difficult temperament, emotional reactivity and impulsivity. According to diathesis-stress models of the aetiology of BPD, these inherited or biological vulnerabilities (e.g. interpersonal hypersensitivity) interact with environmental stressors to increase the risk of the expression of BPD symptoms [12, 13]. Maladaptive parenting, that is, maltreatment, abuse or neglect inflicted on a child by their caregiver, is one environmental stressor that has historically gained attention in literature exploring the aetiology of BPD [13, 14, 15] and has been hypothesised to mediate the association between BPD symptoms in parents and their offspring [16].

Maladaptive parenting has been found to predict BPD features and diagnosis in later adolescence and adulthood [15], with up to 84% of people with BPD retrospectively describing experiences of bi-parental neglect and emotional abuse before the age of 18 [17]. Additionally, maladaptive parenting is thought to contribute to disturbances in emotion co-regulation [18] and the transmission of social knowledge [19], which result in deficits in core psychological processes such as emotional regulation and social cognition [20]. These early experiences of maltreatment, abuse and neglect, and their resulting psychological deficits, place a child at risk of developing a clinical disorder, such as BPD, in adolescence and adulthood. Having a history of maltreatment also appears to influence individuals with BPD in their own parenting role. In a recent study [21] of youth brought to the attention of protective services for history of maltreatment, substance abuse and conjugal violence, 34.3% of mothers had a previous diagnosis or met criteria for BPD. Notably, 50% of mothers with BPD had experienced childhood maltreatment that was severe enough to be reported and as such, were also followed by youth protective services. The parent-offspring relationship therefore appears to be an important context for understanding not only the aetiology of BPD but also how the disorder transmits across generations.

For parents with personality disorder, maladaptive parenting may be related to the additional stress and lower self-efficacy and fulfilment these parents experience compared to those with other mental illnesses [22, 23]. Parents with personality disorder may also struggle with expressing appropriate empathic responses, fluctuations in mental wellbeing, difficulty maintaining a stable and safe environment, role confusion, managing interpersonal conflict, engaging in parenting skills and demonstrating self-efficacy [8]. Mothers with BPD are considered particularly at risk. Maternal BPD is associated with lower sensitivity, emotion recognition, parenting satisfaction and efficacy, and higher intrusiveness, over- protection, hostility, and parenting stress/distress, compared to maternal depressive disorder, other personality disorders and healthy controls [22–26]. Parental personality disorder also places children at risk for a range of emotional and behavioural problems [27]. Children of mothers with BPD display significantly more emotional and behavioural problems than children of mothers with depression only, children of mothers with no psychiatric condition, or children of mothers with ‘cluster C’ personality disorders [8]. More specifically, research suggests that these children experience increased emotional dysregulation, suicidal ideation, insecure attachment styles, depressive symptoms, externalising problems and interpersonal difficulties, as well as poorer general psychopathology and less stable self-image [8, 28–34]. These findings suggest that there may be a sequential relationship between parental personality disorder symptoms, maladaptive parenting and children’s emotional and behavioural problems, and that this relationship may place children at risk of developing BPD in adolescence or early adulthood [35].

There is growing concern for the implications of BPD on parent and child outcomes [36]. However, this area of research is in its relative infancy, and as such, it is crucial that ongoing research utilising robust study methodology is conducted to better understand the role of parenting in both the aetiology and transmission of personality disorder. There has been some attempt to non-systematically review this literature [27], or systematically review parts of the field by exploring psychosocial risk factors for BPD [37] or the impact of maternal BPD on parenting and offspring outcomes [16]. However, the foci of these reviews and the sample population vary, as do their study design, methodology and quality assessment. In order to move the field forward, there is a need for a comprehensive overview on the state of the literature concerning the relationship between parenting and personality disorder: identifying the relevant research, assessing the methodological quality, summarising the findings, and comparing and discussing the strengths of the conclusions. Thus, the primary aim of this overview was to systematically review and qualitatively synthesise published systematic reviews exploring the association between parenting and personality disorder. We chose personality disorder (rather than focusing on borderline personality specifically) to reflect the dimensional approach to classification proposed by the DSM-5 alternate model of personality disorders and ICD-11 [38]. Specifically, we addressed the following research questions:

1. For individuals with personality disorder pathology, what does the research tell us about early exposure to maladaptive parenting and its impact on psychopathological (e.g. prevalence and aetiological) outcomes?
2. For the subset of parents with personality disorder pathology, what does the research tell us about exposure to maladaptive parenting and its impact on relational (e.g. parenting and parent-offspring relationship) outcomes?

## Methods

In conducting our overview of systematic reviews we followed recommendations from the Preferred Reporting Items for Systematic Reviews and Meta Analyses [39] guidelines. A predetermined protocol outlining methods of data searching, inclusion criteria and data extraction method was registered on the International Prospective Register of Systematic Reviews (PROSPERO, registration number: CRD42018096177) available at http://www.crd.york.ac.uk/PROSPERO/display_record.php?ID=CRD42018096177.

### Eligibility criteria

To determine the eligibility of all systematic reviews and primary studies, we followed the PECOS format outlined by PROSPERO. For inclusion in this overview, studies had to meet the following criteria:

1. Participants: Adults aged minimum 18 years with personality disorder pathology (or caring for a child with personality disorder pathology), *and/or* children aged zero to 19 years (unless retrospective measure) with personality disorder pathology (or cared for by a parent with personality disorder pathology). Personality disorder diagnosed or presence of significant symptoms or features detected using a well-validated and structured assessment procedure.
2. Exposure: Maladaptive parenting (including childhood maltreatment, abuse and neglect) measured in individuals with personality disorder pathology either retrospectively or prospectively using well-validated self and other report or observational measures.
3. Comparator(s)/control: Other non-personality disorder mental health condition (PC; psychiatric comparator/control) diagnosed in studies using a well-validated and structured assessment procedure *and/or* participants with no psychopathology (HC; healthy comparator/control) randomly sampled from the community.
4. Outcomes: Studies reported on a range of different primary outcomes pertaining to the relationship between parenting and personality disorder, including psychopathological outcomes (e.g. prevalence and aetiology personality disorder), offspring outcomes (e.g. behavioural, emotional, cognitive), parenting outcomes (e.g. maladaptive parenting including maltreatment, abuse and neglect) and parent-child relationship outcomes (e.g. mother-infant interactions, attachment).
5. Study design: Studies will be included if they are peer-reviewed systematic review articles. We operationally define a systematic review as an overview of a specific research area with robust research methodology (including clear description of the search strategy and methodology) that allows for reproducibility of methodology and findings.

In addition, we also investigated the original primary studies included in the systematic reviews, and extracted data from the studies that met the pre-determined inclusion criteria based on the PECO format (criteria 1–4).

### Study selection

We first performed a systematic search of titles and abstracts in the electronic databases Scopus, Web of Science, MEDLINE, PsycINFO, Psychological and Behavioural Collection to identify peer-reviewed systematic review articles that explored parenting and personality disorder pathology (i.e., diagnosis, symptoms or features), and were published between 1980 and August 2018. We chose 1980 as the lower bound cut-off as this was the year personality disorder was first described as discrete types, grouped into three clusters, and placed on a separate axis (Axis II) in the DSM-III [40]. No language restrictions were applied. We used the following grouped search terms: (“personality disorder” OR “borderline personality disorder” OR “emotionally unstable personality”) AND (mother* OR maternal OR father* OR paternal OR parent*) AND (aetiology OR etiology OR transmission OR pathway OR “risk factor” OR cause OR precursor* OR prodrom* OR antecedent* OR predict*). In addition to electronic database searches, the reference lists of included systematic reviews were hand searched to identify additional sources. The first author (KRS) conducted the initial search. Titles and abstracts of articles identified were screened independently by two authors (KRS, MLT), and then in full. If a title appeared relevant but no abstract was available, the full article was retrieved using the University of Wollongong document delivery service. Full text articles were screened against the eligibility criteria by two authors (KRS, MLT), with a third author and expert in personality disorder research (BFSG) available to help resolve any disagreements.

### Data extraction

We created a data extraction form based on PRISMA and Cochrane guidelines [39] for both systematic reviews and included primary studies. For systematic reviews, we collected information on author, date and country of study, sample, aims, research questions, inclusion criteria, search criteria, study selection process, quality appraisal, major findings and limitations. For each systematic review, we extracted all included primary studies and evaluated whether they matched the eligibility criteria for this overview based on the PECO format outlined above. For the primary studies that met the inclusion criteria, we collected information on first author, date and country of study, personality disorder construct, assessment tool, setting, study design and participant demographics (i.e. number of participants, clinical groups, gender, age and race).

### Assessment of methodological quality

Two independent raters (KRS and MLT) assessed the methodological quality of each included systematic review using the Critical Appraisal Skills Programme Systematic Review Checklist [41]. The CASP Systematic Review Checklist is a 10-item tool designed to assess the methodological quality of a systematic review. The CASP considers three broad areas when appraising a systematic review: are the results valid; what are the results; will the results help locally?

### Data synthesis

Due to the heterogeneity of systematic reviews and primary studies (including differences in primary study designs, participants, settings, personality disorder pathology and measurement tool and outcomes of interest), a meta-analysis of results was not feasible. However, when meta-analysis was performed in the systematic reviews, we reported on the pooled estimates described by the authors using 95% confidence intervals. Systematic reviews were qualitatively synthesised using a narrative review and the text analysis software package Leximancer (version 4, 2011). We used Leximancer to identify the most common themes, concepts and relationships in the systematic reviews, depicted through a visual map. On the map, the proximity of concept dots represents their relatedness and the size of the concept dot represents how frequently concepts are presented in the text. Leximancer software was initially used to conduct an automatic text analysis of the included systematic reviews. Following this, we refined results by grouping words (e.g. “parent” and “parenting”) and removing irrelevant common words. The minimisation of subjectivity in the analytic process was ensured through discussion among the research team about emerging themes, where any discrepancies were resolved via consensus.

## Results

**Fig 1:**
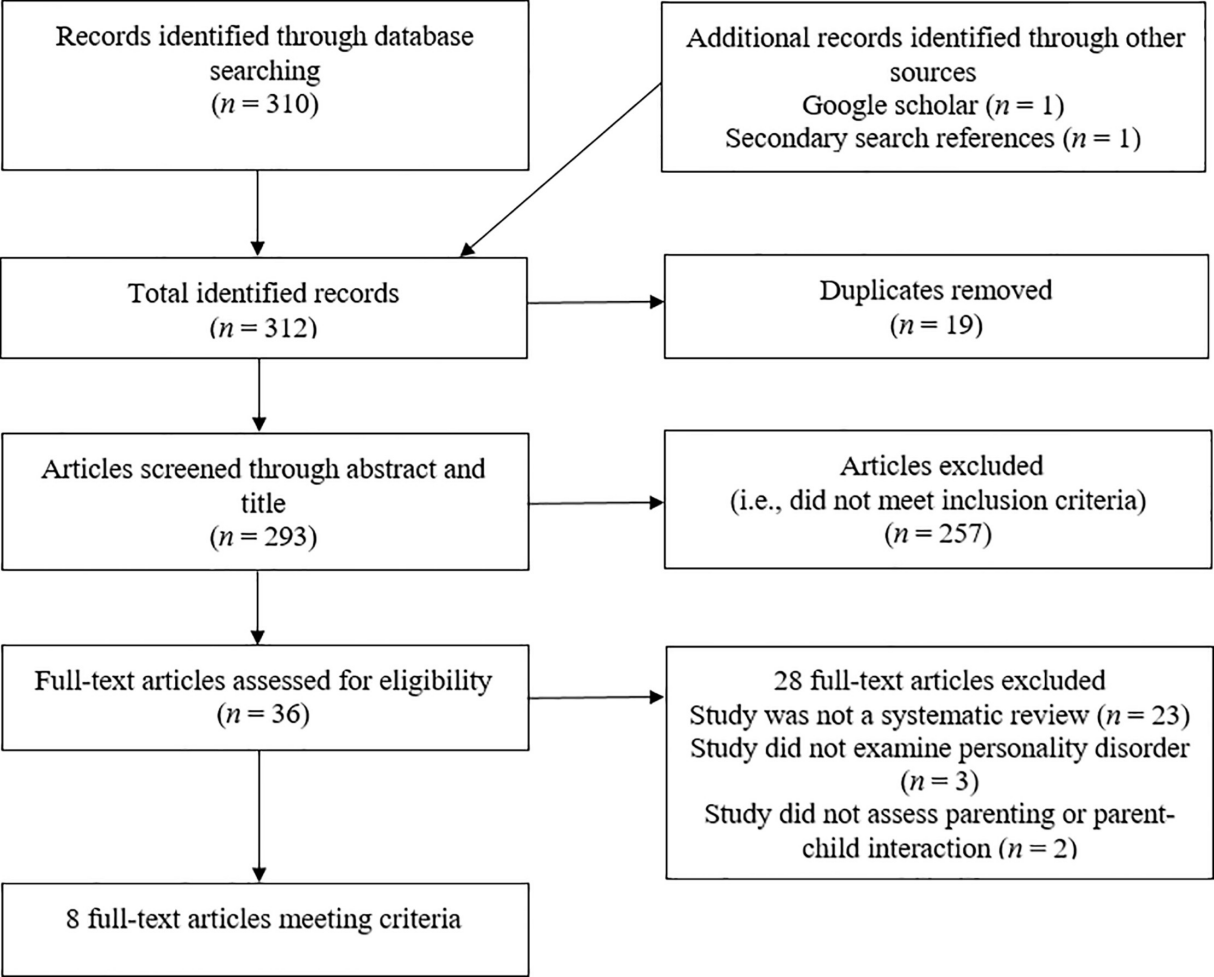
Preferred reporting items for systemic reviews flow diagram of search and selection of systematic reviews included in our overview.

A total of 312 sources were identified through electronic database searching (*n* = 310) and identifying additional sources (*n* = 2). After the removal of 19 duplicates, 293 sources were screened through their title and abstract. We excluded 257 citations that did not meet the inclusion criteria, leaving 36 sources eligible for full-text retrieval, with an excellent level of agreement between independent raters (Cohen’s Kappa; К = .90; *p* < .001). Of these sources, 28 sources were excluded due to not using a systematic methodology (*n* = 23), not studying personality disorder (*n* = 3) and not studying parenting or parent-child interactions (*n* = 2). Eight full-text articles met all criteria for inclusion in the review. Independent raters were in perfect agreement for final inclusion for the review (К = 1.00; *p* < .001). Within the eight included systematic reviews, there were 211 primary studies, of which 140 (66.35%) met all criteria for inclusion in the review. For a flowchart outlining the search and selection of studies, see Fig 1.

### Methodological quality

The quality of systematic reviews was assessed using the CASP Systematic Review Checklist (S1 Table). Five systematic reviews reported assessing the quality of their included studies. In one review the authors developed a quality assessment based on the CASP [42], whilst the remaining studies used established quality assessments including the STROBE checklist [43], Newcastle-Ottawa Scale [16, 44] and CASP for case-control studies [45]. Only studies that the authors deemed to have medium-high methodological quality were reported. Notably, three systematic reviews did not report quality assessment [37, 46, 47], and as such their methodological quality is unclear. Additionally, five systematic reviews did not have a clearly defined research question [37, 42, 44–46], two systematic reviews conducted their searches in only two databases [43, 46], two did not conduct hand searches [46, 47] and one did not detail a study selection process [43]. Despite these limitations, independent raters agreed that all studies had sufficient methodological quality to be included in the review, meaning their results could be generalised and applied to the study population, with an excellent level of agreement between independent raters (К = .94; *p* < .001).

### Study characteristics

#### Systematic reviews

Eight systematic reviews explored the relationship between parenting and personality disorder [16, 37, 42–47] (Table 1). Included systematic reviews were published in the United Kingdom (*n* = 5), USA (*n* = 1), Canada (*n* = 1) and Finland (*n* = 1) between 2012 and 2015. The majority of systematic reviews (*n* = 7) focused on BPD specifically, with one reporting on other personality disorders (OPD) [42]. All systematic reviews included a broad aim, search and inclusion criteria, and three included specific research questions [16, 43, 47]. The number of primary studies included in the systematic reviews ranged from 10 [45] to 61 [44]. The study selection process was outlined by all but one systematic review [46], with two adopting the PRISMA protocol [16, 37] and five including a quality appraisal assessment [16, 42–45]. The types of studies eligible for inclusion varied amongst the systematic reviews, with five including longitudinal designs [16, 37, 44, 45, 47], four including case-control designs [16, 42, 45] and six including cross-sectional designs [16, 42–45, 47]. We found five systematic reviews reported on maladaptive parenting (including childhood maltreatment, abuse and neglect) as a psychosocial risk factor for the development of BPD [37, 44–47], two reported on the impact of BPD on mother and offspring outcomes [16, 43] and one reported on the impact of personality disorder on parent and offspring outcomes [42]. All but one systematic review [44] reported qualitative synthesis only. The most common reason for not conducting meta-analysis was heterogeneity of primary study designs.

**Table 1:** Study characteristics of the included systematic reviews.

Author (date) Country	Keinan, et al. (2012) Finland	Laulik, et al. (2013) UK	Petfield, et al. (2015) UK	Eyden, et al. (2016) UK	Stepp, et al. (2016) USA	Winsper, et al. (2016) UK	Boucher, et al. (2017) Canada	Ibrahim, et al. (2018) UK
Review Type	Systematic review and qualitative synthesis	Systematic review and qualitative synthesis	Systematic review and qualitative synthesis	Systematic review and qualitative synthesis	Systematic review and qualitative synthesis	Systematic review and meta-analysis	Systematic review and qualitative synthesis (narrative review)	Systematic review and qualitative synthesis
Title	A systematic review of the evidence-based psychosocial risk factors for understanding of borderline personality disorder	The link between personality disorder and parenting behaviours: A systematic review	Parenting in mothers with borderline personality disorder and impact on child outcomes	A systematic review of the parenting and outcomes experienced by offspring of mothers with borderline personality pathology: Potential mechanisms and clinical implications	A systematic review of risk factors prospectively associated with borderline personality disorder: Taking stock and moving forward	The aetiological and psychopathological validity of borderline personality disorder in youth: A systematic review and meta-analysis	Parent-child relationship associated with the development of borderline personality disorder: A systematic review	Childhood maltreatment and its link to borderline personality disorder features in children: A systematic review approach
Sample	51 case-control and cohort studies examining psychosocial vulnerability factors for BPD that met the international EBM criteria.	11 case-control, cohort and cross-sectional studies that examined parents with PD pathology (i.e. PD dx or significant features).	17 cross-sectional studies that assessed mothers with BPD dx who were the primary caregiver to child/children (under 19 years).	33 case-control, cross-sectional and cohort quantitative studies that examined mothers with BPD pathology and/or children of mothers with BPD pathology.	39 longitudinal, prospective studies, exploring risk factors associated with BPD dx, sxs and features in 43,681 mainly female (54%) and Caucasian (69%) participants in community samples (73%).	61 retrospective, cross-sectional and prospective studies that examined the aetiological and psychopathological validity of youth BPD (19 years and under).	40 mostly cross-sectional (two longitudinal) studies that presented the perspectives of individuals with BPD and their parents and families	10 longitudinal cohort, case-control and cross-sectional studies that explored the association between maltreatment and BF in children (12 years and under).
Aims	To provide a systematic review of the literature focussing on the psychosocial risk factors for BPD.	To determine if parental personality disorder is associated with impaired parenting practices and poor quality of parent-child interactions, and to provide directions for future research.	To systematically synthesise research findings in order to provide a better understanding of the consequences of maternal BPD.	To systematically search and narratively synthesise all research examining the parenting behaviours and attitudes of mothers with BPD, mother-offspring interactions, and offspring outcomes.	To summarise and synthesise identified study results, detail risk factors and discuss strengths and limitations of the literature. Secondly, to determine if evidence regarding BPD risk factors enhances developmental theories by explaining aetiology and identifying those at risk.	To examine associations between psychopathological and aetiological factors identified in the literature on adult and youth BPD. Secondly, to examine associations with continuous BPD symptoms in adults and youth.	To synthesise all relevant studies on PCR in BPD from 1980 onwards.	To explore research looking at associations between maltreatment and BPD or BF in childhood
Research Questions	N/R	N/R	1. Are there deficits and difficulties in the parenting of mothers with BPD? 2. What difficulties are experienced by children of mothers with BPD?	1. What are the characteristics parenting behaviours of mothers with BP pathology? 2. How do mothers with BP pathology and their offspring interact? 3. What are the psychopathological and psychological outcomes for offspring of mothers with BP pathology? 4. What are the mechanisms underpinning associations between maternal BP pathology and offspring outcomes?	N/R	N/R	1. How is PCR described by BPD participants and their parents in comparison to other normative and clinical groups? 2. Which aspects of the PCR are specifically associated with a BPD diagnosis in adulthood? 3. How can the facets of the PCR identified in the reviewed studies shed light on the general aetiological models of BPD?	N/R
Inclusion Criteria	1. Well documented cohort or case and control group studies that use the international ICD-10 or DSM-IV diagnostics, well documented standard patient interview methods and reliable statistical evaluations	1. Parents aged 18 years or over PD diagnosed using a structured assessment procedure or presence of significant PD features 2. Assessment of parenting behaviours to include the quality of observed parent-infant interactions, self-reported parenting behaviours and recorded incidents of child abuse or maltreatment 3. Cohort, case control studies, case-series or cross-sectional studies 4. All languages	1. Mothers diagnosed with BPD using standardised assessment procedures (or diagnostic techniques based on earlier editions of the DSM for older studies) 2. Mothers must be the primary caregiver to their child/children 3. Mothers aged 18 or over. 4. Children aged 18 or under 5. Studies must measure factors influencing the mother’s parenting and/or her child’s functioning 6. Studies must be written in English 7. Studies must present outcome data 8. Studies must be from peer-reviewed journals 9. Studies must be quantitative in design	1. Mother BPD dx or sxs and/or offspring (of any age) of mothers with BPD dx or sxs (assessed via standardised measure) 2. Maternal parenting, and/or offspring outcomes reported on using a range of assessment methods 3. Samples consisted of mainly mothers (i.e. at least 70%)	1. Prospective, longitudinal studies of any follow-up duration with at least two assessment points 2. Outcome included BPD features, sxs, or dx 3. Risk factor was measured prior to BPD outcome assessment	1. Study published in a peer reviewed journal 2. Participants were 19 years or under at index assessment 3. Study published in English 4. Study provided information on either psychopathological or aetiological correlates of youth BPD 5. Study reported OR and CI comparing those with to those without BPD dx or sxs or provided information that could be used to calculate OR and CI	1. Study focused on the PCR in BPD Study compared BPD participants perception and/or parents of BPD participants perception of PCR to at least another control group or predicted BPD diagnosis with PCR measure 2. Study presented a valid instrument for confirming BPD dx in late adolescence or adulthood 3. Study included participants with BPD dx 4. For cross-sectional studies, included a sample composed of mostly adult participants (aged 18 and over) 5. Data collected directly from the BPD participants and/or their parents 6. Results presented specifically address parent-child interactions (measured in at least one other screened article)	1. Study makes an association of any type of maltreatment with BF in children or children with BPD dx 2. Study used case-control, cross-sectional and longitudinal cohort design 3. Study published in peer-reviewed journals 4. Study published in English
Search Criteria	Searched were conducted in the electronic databases Medline and PsychInfo using the search terms ‘borderline personality disorder’ and ‘risk factors’.	Searches were conducted in the electronic databases PsychINFO, Medline, EMBASE and Web of Science using an unknown search string that covered the following concepts: personality disorder, parenting capacity, parenting efficacy, parenting behaviours, parent-child interactions, child abuse and neglect; yielding 15,039 articles. 22 studies were identified through experts and two were hand searched from reference lists.	Searches were conducted in the electronic databases PsycINFO and MEDLINE using the search string: “child*” AND (“borderline personality disorder” OR “emotionally unstable personality disorder”); yielding 3814 articles. No hand searched articles were included.	Searches were conducted in the electronic databases PsychINFO, PubMed, EMBASE, Web of Science, Scopus and ASSIA using the search terms (borderline* or “emotionally unstable personality” or BPD) and (mother* or parent* or maternal*) and (child* or infant* or infancy or offspring or bab* or adolescen* or famil* or boy* or girl* or teenager* or youth* or young* or toddler* or daughter* or son*); yielding 10,047 articles. An additional 21 articles were extracted from hand searching.	Searches were conducted in electronic databases PubMed, CINAHL, PsychINFO, and ISI Web of Science using the search terms (borderline personality and [longitudinal or follow-up* or prospect*] and [precursor* or risk factor* or prodrom* or antecedent* or predict*] and [diagnosis or development]) and using hand searches of reference lists; yielding 376 results.	Searches were conducted in electronic databases Medline, Embase, PsychInfo and PubMed using the search string: (borderline* or “emotionally unstable personality disorder” or BPD) and (adolescen* or child* or young* or teen* or student*); yielding 19,078 articles. An additional 4 articles extracted via hand searching of reference lists of included studies and relevant narrative reviews.	Searches were conducted in the electronic databases PsychINFO, Medline and Web of Science using the search terms ‘borderline personality disorder’ and (mother* or father* or caregiver* or caretaker* or parent*) and the limits peer-reviewed journals, English or French language and earliest publication year 1980; yielding 1277 articles. No hand searched articles were included.	Searches were conducted in the electronic databases OvidSP, PubMed and Scopus using the search terms maltreatment, borderline disorder or borderline features and child; yielding 3902 results. The online database PsycINFO was used to map the primary search term, borderline and child and features or state or personality or traits or disorder and maltreatment or physical abuse or sexual abuse or verbal abuse or emotional abuse or neglect or foster or in care or looked after or adopted or institution or children’s home. Google scholar was used to check for unidentified articles using the search term borderline and children; yielding 211 results. An additional three articles were identified through hand searching reference lists.
Study selection	N/R	1. Duplicates and ‘irrelevant’ articles were excluded, leaving 250 articles 2. 229 were removed in accordance with the inclusion/exclusion criteria, one primary study was unattainable and one could not be located 3. 19 studies underwent quality assessment, in which eight were determined to be poor quality, ten moderate quality and one high quality. 4. 11 primary studies were eligible for review	1. Non-English articles and duplicates were removed, leaving 2579 articles 2. These title and abstracts of articles were screened against inclusion/exclusion criteria by 1^st^ author (10% were re-rated by an independent researcher with an agreement of 97.8%) 3. 70 articles were eligible for full text retrieval and screened against the inclusion/exclusion criteria 4. 17 primary studies were deemed eligible for review	Study selection was based on the PRSIMA and Cochrane guidelines: From abstract screening, 101 articles were identified for full-text retrieval. The level of agreement between 1^st^ and 2^nd^ author was ‘excellent’ (К = .87, *p* < .001) After screening full-text articles, 33 studies were deemed eligible for inclusion in the review. Inter-rater agreement for final inclusion for the review was ‘excellent’ (К = .88, *p* < .001)	Study selection was based on PRISMA-P guidelines: 1. All studies were screened based on title and abstract, resulting in 311 studies being excluded 2. 65 full-text articles were reviewed against the inclusion/exclusion criteria 3. Authors performed independent evaluations resulting in removal of 26 4. 39 primary studies were deemed eligible for review	Duplicates were excluded, leaving 8195 articles All titles and abstracts were scanned by the first two authors, resulting in 7986 records being excluded. There was a high level of agreement between 1^st^ and 2^nd^ raters (К = .82) 213 articles (209 initial search and 4 hand search) were identified for full-text retrieval. The 1^st^ author read relevant articles identified for full text retrieval and assessed for their inclusion in the review. The 3^rd^ author independently reviewed 50% of full text articles for inclusion in the final review as a reliability check (К = .80) 61 articles were deemed eligible for review	1. Duplicates were excluded, leaving 814 articles. All titles and abstracts were reviewed independently by two researchers 2. Of the remaining articles, 729 were removed in accordance with the inclusion/exclusion criteria, leaving 85 articles eligible for full text retrieval 3. Of these articles, 45 were removed due to the inclusion/exclusion criteria 4. 40 articles were deemed eligible for review	1. Any articles that did not meet the study criteria were removed, included duplicates 2. From remaining titles, abstracts were retrieved and read 3. For studies meeting inclusion and exclusion criteria, the full text article was retrieved 4. If the independent rater agreed on the quality rating the study was included and the references of the full-text articles were manually screened to identify any further relevant articles
Quality Appraisal	N/R	Quality assessment was formulated based on the CASP. To ensure consistency in the quality of the studies assessed, a 2^nd^ independent reviewer verified 20% of the studies. Only studies deemed to be of moderate to high quality (i.e. 70% of above) were included in the review.	Quality was appraised using the STROBE checklist. On a scale of zero (bad) to five (good), four papers were categorised as ‘average to above average’ and 13 as ‘above average to good’. Five randomly selected articles were rescored by an independent rater, with inter-rate reliability giving a strong positive correlation (*r*_s_ = 0.95, n = 5, *p* = 0.014).	Quality was assessed by the 1^st^ author using the Newcastle-Ottawa Scale, with the 2^nd^ author independently assessing 50% of the studies for reliability. The quality assessment showed substantial inter-rater agreement (К = .77, *p* < .001) and indicated low risk of bias in sample section, low risk of comparability bias and low-moderate risk of exposure/outcome bias.	N/R	A quality assessment tool based on the Newcastle-Ottawa Scale was used to rate the methodological quality of each study. Each study was given a total score (out of six for aetiological factors, and five for psychopathological factors) reflecting key aspects of study methodology.	N/R	Quality was assessed using the CASP for case-control studies. The CASP considers three broad areas: are the results valid; what are the results; will the results help locally?
Reported Findings	Five vulnerability factors were identified and classified according to the EBM-criteria of best evidence: 1. Childhood trauma/abuse 2. Unfavourable parenting 3. Object relations 4. Insecure attachment/loss 5. Symbolisation-reflectiveness capacity	Nine studies found evidence to support the existence of a positive association between PD dx and features and impaired parenting behaviour. In these studies, the presence of PD was related to: • The use of inadvisable and problematic parental practices • Inconsistent parental discipline • Low parental affection, assistance, praise and encouragement • Less satisfaction and reported competence in the parenting role • Insensitive, instructive poorly attuned and disrupted parent-infant interactions • Harsh behaviour • Frightening/disoriented parental behaviour • Status as an abusive parent	Maternal BPD dx was associated with differences in parenting outcomes compared to control group, including: • Reduced sensitivity and increased intrusivity towards child • Difficulty with unstructured activities and having poorer levels of family organisation • Family environments characterised by high levels of hostility and low levels of cohesion • Increased overprotection • Poor mind-mindedness • Less competence and satisfaction in parenting role • Increased parenting stress Maternal BPD dx was also associated with differences in children’s outcomes compared to control groups, including: • Less satisfying interactions • More cognitive-behavioural risk factors • Difficulties in mother-child relationship • Poorer mental health	Compared to control groups, mothers with BPD dx or sxs appear: • Less sensitive • Less engaged • More intrusive • More overprotective • More hostile • More likely to have maladaptive interactions with their offspring Offspring exhibited a range of psychological (e.g. BPD dx, sxs and features, depression, internalising/externalising problems, general psychopathology, and psychosocial (e.g. poor self-esteem, interpersonal difficulties, home difficulties, general impairment) outcomes across several stages of development. Potential mechanisms underpinning the transmission of vulnerability from mother to offspring include: Maladaptive parenting Maternal emotional dysfunction Offspring characteristics	Multiple factors across social, familial, maltreatment and child domains increase the risk for BPD. The most robust risk indicators in there domains were: • Social: low SES, stressful life events, family adversity • Family: maternal psychopathology, affective parenting dimension (e.g. low warmth, hostility, harsh punishment) • Maltreatment: physical or sexual abuse, neglect • Child: low IQ, negative affectivity and impulsivity, internalising and externalising psychopathology	Adult and youth BPD share common aetiological and psychopathological correlates: • Statistically significant pooled associations (OR [95% CI]) with all youth BPD were observed for sexual abuse (4.88 [3.30, 7.21]), physical abuse (2.79 [2.03, 3.84]) maternal hostility/verbal abuse (3.28 [2.67, 4.03]) and neglect (3.40 [2.27, 5.11]) • Several psychopathological features were also associated with youth BPD, including comorbid mood (3.21 [2.13, 4.83]), anxiety (2.30 [1.44, 3.70]), and substance use disorders (2.92 [1.60, 5.31]), self-harm (2.81 [1.61, 4.90]), suicide ideation (2.02 [1.23, 3.32]), and suicide attempt (2.10 [1.21, 3.66]	BPD participants and their parents consistently reported a more dysfunctional PCR compared to PC and HC: • BPD participants report lower parental care and higher parental overprotection and inconsistency • Parents retrospectively describe their BPD child as being unusually sensitive, having a ‘difficult temperament’ and their relationship as marked by verbal abuse and violent and antisocial behaviours • Family perspective studies suggest that BPD daughters report less parental care, more maternal overprotection and inconsistent parental values and norms • BPD and Axis-I participants are discriminated by lack of parental care, inconsistency, abuse and neglect BPD and Axis-II outpatients are discriminated by rates of parental abuse, neglect, care, overprotection and inconsistency	There is a link between maltreatment and BF in childhood: • Children with BF were more likely to have a history of maltreatment compared to PC • Maltreated children compared to non-maltreated child were more likely to present with BF Other risk factors (e.g. deficits and cognitive and executive functioning, parental dysfunction and genetic vulnerability) were also identified.
Limitations	1. The identified risk factors are not independent of each other 2. Only psychosocial risk factors are delineated 3. Subcategories of abuse/neglect are not examined separately	1. The review comprised of only a small number of studies of varied quality 2. Confounding risk factors were not included or explored in sufficient detail 3. BPD was the specific focus of 4 of 11 studies. Therefore, the findings of the review may not be generalisable to OPD 4. Three studies considered the impact of paternal PD, with no studies specifically examining father-infant interaction. Therefore, findings may not be generalised to fathers with PD In the mother-child interaction studies, infants were the primary focus with only 1 study exploring interactions between older children and their mothers. Therefore, findings may not be generaliseable to older children and adolescents	1. The review excluded all papers that were not in English 2. All included studies were cross-sectional 3. In some cases, BPD dx was achieved through self-report questionnaire only 4. Parents were often the primary reporter on children’s outcomes 5. Almost all included studies were very small and underpowered to detect small group differences, impacting generalisability of findings and increasing the risk of publication bias 6. Most of the included studies employed clinical samples, which may be over-representative of the severe presentations	1. Heterogeneity in the operationalisation of parenting constructs, offspring outcomes and study method design 2. Participant selection criteria differed across studies 3. Assessment of mother’s BPD dx differed across studies. 4. Age of offspring varied, with some samples crossing developmental stages 5. Insufficient number of studies to make inter-study comparisons or draw firm conclusions in some domains 6. Quality assessment showed a low-moderate risk of outcome/exposure bias and publication bias due to the “file drawer” problem 7. The review excluded child outcomes that required external intervention 8. Majority of studies were cross-sectional	1. There is a lack of specificity, with previous research demonstrating a nearly identical risk profile for a broad range of internalising and externalisation disorders 2. There was a degree of heterogeneity across several study features, with only 24 of 39 included studies representing unique samples	1. Results may have been subject to publication bias due to the “file drawer” problem 2. Analysis is affected by the broadness of assessments for risk and psychopathological factors, and a lack of consistency in tools across studies 3. Assessments of BPD varied across studies 4. Variation in controlling of confounding variables resulted in unadjusted associations being meta-analysed. The extent to which associations may have been reduced by confounding variables is unknown. 5. Not all relevant psychopathological and aetiological factors could be quantitatively synthesised	1. Cross-sectional studies on children or adolescents and all studies on mothers with BPD and their own children were excluded. This meant that a dimensional evaluation of BPD was not possible 2. Results can only be generalised to relationship between adults/late adolescents with BPD (evaluated categorically) and their parents 3. Some results reported by a single study only 4. Heterogeneity of variables measured, instruments used and perspectives taken by the studies reviewed lead to contradictory results.	1. Studies used different methods to diagnose BPD or identify BF, with some not yet validated and others subject to informant bias 2. Studies used different definitions and classifications of abuse/neglect 3. Studies did not consider the severity of maltreatment and how this may impact BF 4. The review used the search term ‘borderline features’, which may not have been applicable to early research

*Note*. BPD = Borderline Personality Disorder. EBM = Evidence Based Medicine. PD = Personality Disorder. dx = diagnosis. sxs = symptoms. BF = Borderline Features. PCR = Parent-Child Relationship. ICD-10 = International Statistical Classification of Diseases and Related Health Problems– 10^th^ revision. DSM-IV = Diagnostic and Statistical Manual for Mental Disorders– 4^th^ Edition. HC = Healthy Comparison/control. OR = Odds Ratio. CI = Confidence Interval. PC = Psychiatric Comparison/control. PRISMA = Preferred Reporting Items for Systematic Reviews and Meta-Analyses. CASP = Critical Appraisal Skills Programmes. STROBE = Strengthening the Reporting of Observational studies in Epidemiology. SES = Socioeconomic Status. IQ = Intelligence Quota. OPD = Other Personality Disorders.

#### Primary studies

Within the eight systematic reviews, there were 211 primary studies of which 140 (66.35%) met the eligibility criteria for this overview. The eligible primary studies were predominately published in North American (USA: *n* = 74; Canada: *n* = 21), United Kingdom (*n* = 11) and Australia (*n* = 8), between 1985 and 2015. The vast majority of primary studies focused on BPD diagnosis, symptoms or features specifically (*n* = 100), with an additional 39 reporting on BPD and other personality disorders, symptoms or traits (e.g. Narcissistic personality disorder; NPD) and one reporting on NPD characteristics only. BPD was predominately assessed using the Structured Clinical Interview for DSM Axis II disorders (SCID-II: *n* = 47), the Diagnostic Interview for Borderlines (DIB: *n* = 44), and/or DSM criteria based psychiatric evaluation (*n* = 19). The primary studies utilised cross-sectional (*n* = 29), case-control (*n* = 62) and longitudinal cohort (*n* = 49) designs, in clinical inpatient (*n* = 41), outpatient (*n* = 77), and/or community settings (*n* = 102). Of the 140 included primary studies, 120 (85.71%) were drawn from unique data sets, with the remainder (*n* = 20) reporting a common data set. For example, Avon Longitudinal Study of Parents and Children (ALSPC), Children in the Community Study (CIC), Greifswald Family Study (GFS), Study of Health in Pomerania, Germany (SHIP), Mater University Study of Pregnancy (MUSP), and Pittsburgh Girls Study (PGS). There was some overlap in studies included in the systematic reviews, with 42 (30%) of the eligible primary studies included in two or more reviews.

There were 121,895 participants across all included primary studies, of whom 84,333 (69.18%) were uniquely sampled. Adult participants were utilised by 58 studies (S2 Table), child and adolescent participants by 28 (S3 Table) and both adult and child participants by 54 (i.e. parent-offspring studies; S4 Table). There were 14,167 adult participants, approximately 73% female, 79% Caucasian, 31.99 ± 7.06 (18.93–45.92) years, of whom 3,909 (27.59%) received a research or clinical diagnosis of BPD or reported clinically relevant BPD symptoms. Additionally, there were 9,686 child and adolescent participants, approximately 63.13% female, 51.5% Caucasian, 13.8 ± 1.38 (9.37–14) years, of whom 919 (9.49%) were given a research or clinical diagnosis of BPD or reported clinically relevant BPD symptoms. There were also 49,079 parent participants, approximately 94.52% female, 61.15% Caucasian, 34.17 ± 5.42 (22.8–47.88) years, and 48,963 offspring participants. Offspring included 14,686 infants, approximately 52.63% female, N/R% Caucasian, 10 ± N/R (10–33) months, 1,801 children, approximately 41.91% female, 80% Caucasian, 6.94 ± 1.41 (2.79–5.80) years, 27,856 adolescents, approximately 69.18% female, 50% Caucasian, 14.74 ± 1.63 (13–16.9) years, and 4,620 adults, approximately 66.78% female, 87.8% Caucasian, 23.48 ± 2.97 (18.29–31.29) years. In these studies, 790 (1.61%) parents, 150 (0.54%) adolescent and 135 (2.92%) adult offspring received a research or clinical diagnosis of BPD or reported clinically relevant BPD symptoms.

### Outcomes

#### The relationship between maladaptive parenting and the aetiology of personality disorder pathology

Five systematic reviews explored the role of maladaptive parenting practices as a psychosocial risk factor for the development of personality disorder [37, 44–47]. The first identified study published by Keinanen et al [46] systematically reviewed 51 case-control and cohort studies examining psychosocial vulnerability factors for BPD that met the international evidence-based medicine (EBM) criteria. The authors identified and classified five vulnerabilities factors for the aetiology of BPD, two of which related to childhood trauma/abuse (risk factor 1) and “unfavourable” parenting (risk factor 2). However, these risk factors are not independent of each other (e.g. unfavourable parenting may include abuse), and childhood trauma/abuse includes serval subtypes of abuse and neglect that were not delineated in this review. Moreover, we found that the authors did not include a specific research question, study selection process or methodological quality assessment, and as such, results of this study should be interpreted with caution. Psychosocial risk factors for BPD were also investigated by Stepp et al. [37], who systematically reviewed 39 longitudinal, prospective studies that incorporated 43,681 mainly female (54%) and Caucasian (69%) participants from community samples (73%). The authors identified family (namely parent/family psychopathology, parenting behaviour/style and family climate and parent-child relationship), and maltreatment and other trauma as two risk factors prospectively associated with BPD. Within these domains, maternal psychopathology, affective parenting dimension (i.e. low warmth, hostility, and harsh punishment) and exposure to physical or sexual abuse/neglect were identified as the most robust risk factors. Of note, we found that this study did not report a clearly defined research question or a methodological quality appraisal, and that many of the included studies that found a positive link between maltreatment and BPD were conducted using the CIC cohort [48–55]. Moreover, whether these psychosocial risk factors are unique to BPD or are representative of vulnerability to mental illness more generally remains unclear. Lack of quality appraisal and oversampling may have resulted in sampling bias, and consequently impact the accuracy and generalisability of the findings.

Childhood maltreatment and its association with borderline features in children (12 years and under) was investigated by Ibrahim et al [45]. By systematically reviewing 10 longitudinal cohort, case-control and cross-sectional studies, the authors concluded that in general, maltreatment is a risk factor for borderline features in children and adults, and that risk is increased by the severity of abuse. However, there was inconsistency in the definition and classification of child maltreatment and a lack of research delineating the effect of different types of abuse and neglect. Due to the heterogeneity of studies, their reliance on self-report and subjective measures that have not yet been adequately validated in the literature, the validity of these findings is uncertain. Childhood maltreatment (including sexual and physical abuse, maladaptive parenting, neglect and parental conflict) was also found to be an aetiological risk factor for BPD diagnosis in children and adolescents by Winsper et al. [44]. In a systematic review of 61 retrospective, cross-sectional and prospective studies, the authors found that the greatest psychosocial risk factor for the aetiology of BPD was sexual abuse for children and parental conflict for adolescents. However, the authors note that not all relevant articles could be quantitatively analysed and thus many potentially relevant aetiological factors (i.e. biological predisposition and insecure attachment) were not included in this systematic review. Additionally, there was heterogeneity in the way the included primary studies controlled for confounding variables and as such, the accuracy of reported associations is unclear.

The parent-child relationship and its association with the development of BPD was investigated by Boucher et al. [47] in their systematic review of 40 mostly cross-sectional studies (*n* = 38) that explored the perspectives of individuals with BPD, and their parents and families. The authors found that individuals with BPD reported lower parental care and higher parental overprotection, parental inconsistency, parental abuse and neglect and negative parental attitudes, with one study finding that maternal overprotection and inconsistency predicted BPD diagnosis [56]. Notably, this systematic review is predominately based on cross-sectional studies utilising a retrospective self and other report, and therefore retrospective bias may be influencing their responses. Moreover, this study does not appear to include a quality assessment, and as such, the methodological quality of included studies is unclear. In light of these methodological limitations, results of this systematic review should be interpreted with caution. Additionally, primary studies reporting on mothers with BPD and their children were excluded from this systematic review and thus, these findings are not applicable to this population.

#### The impact of personality disorder pathology on parent, offspring and parent-offspring relationship outcomes

Three systematic reviews reported on the impact of personality disorder on parenting and offspring outcomes [16, 42, 43]. Through the lens of attachment theory, Laulik et al. [42] explored the link between personality disorder and parenting capacity. The authors systematically reviewed 11 case-control, cohort and cross-sectional studies that examined parents with personality disorder pathology. Nine studies found a positive relationship between maternal personality disorder and maladaptive parenting practices (including child maltreatment), whilst two found neutral or inconsistent results. However, only a small number of studies, which varied in quality (e.g. lack of standardised measures, reliance on observational methods, convenient sampling), were included in this review. Additionally, four studies specifically examined the effect of maternal BPD on parenting variables, particularly within the context of mother-infant interactions. As a result, the findings of this review may not be generalisable to other groups such as fathers with personality disorder, mothers with non-BPD Axis II disorders or older children and adolescents.

Parenting difficulties experienced by mothers with a diagnosis of BPD and their impact on infant and child outcomes were explored by Petfield et al. [43]. In their systematic review of 17 cross-sectional studies, the authors found that maternal BPD was associated with differences in parenting outcomes compared to a control group in a number of different behavioural (e.g. reduced sensitivity), affectual (e.g. increased stress) and cognitive (e.g. poor mind-mindedness or mentalization) domains. Maternal BPD diagnosis was also associated with differences in children’s outcomes compared to a control group, including less satisfying interactions, more cognitive-behavioural risk factors, mother-child relationship difficulties and poorer mental health. Notably, mothers with BPD and their children showed greater difficulty and poorer outcomes compared to parents with other severe presentations (e.g. OPD or Major depressive disorder; MDD). These results are, however, limited by all included primary studies adopting a cross sectional design, heavy reliance on parent-report measures of child outcomes and self-report measures of maternal BPD, utilisation of predominately clinical samples and small sample sizes resulting in a lack of power to detect between and within group differences.

Building on the previous review, Eyden et al. [16] examined the parenting and outcomes experienced by offspring of any age (including adults) of mothers with borderline personality pathology (including diagnosis or symptomology). A systematic review of 33 case-control, cross-sectional and cohort studies found that compared to control groups, maternal BPD was associated with reduced sensitivity, engagement and emotion recognition, and heightened intrusivity, overprotection, hostility. On the other hand, mixed results were found for maternal warmth, rejection and laxness, representations and perceptions of offspring. Mixed results were also found for the impact of maternal BPD on mother-offspring dynamic, particularly in the domain of role-reversal, mother-infant communication and infant/child behaviour. The offspring of BPD mothers did however exhibit a range of psychopathological (e.g. BPD symptoms and related features, depression, internalising/externalising problems, general psychopathology) and psychosocial outcomes (e.g. difficulties with self-esteem, interpersonal difficulties, home stability, general impairment) across several stages of development. The authors proposed that maladaptive parenting is one potential mechanism underpinning the transmission of vulnerability for BPD from mother to offspring. However, in light of the heterogeneity across studies (including study method design, participant selection criteria and assessment of maternal BPD), the scarcity of relevant studies in certain domains (e.g. maternal emotion recognition and rejection), the reliance on cross-sectional study designs and a low-moderate risk of outcome/exposure and publication bias, these results should be interpreted with caution.

### Data synthesis

**Fig 2:**
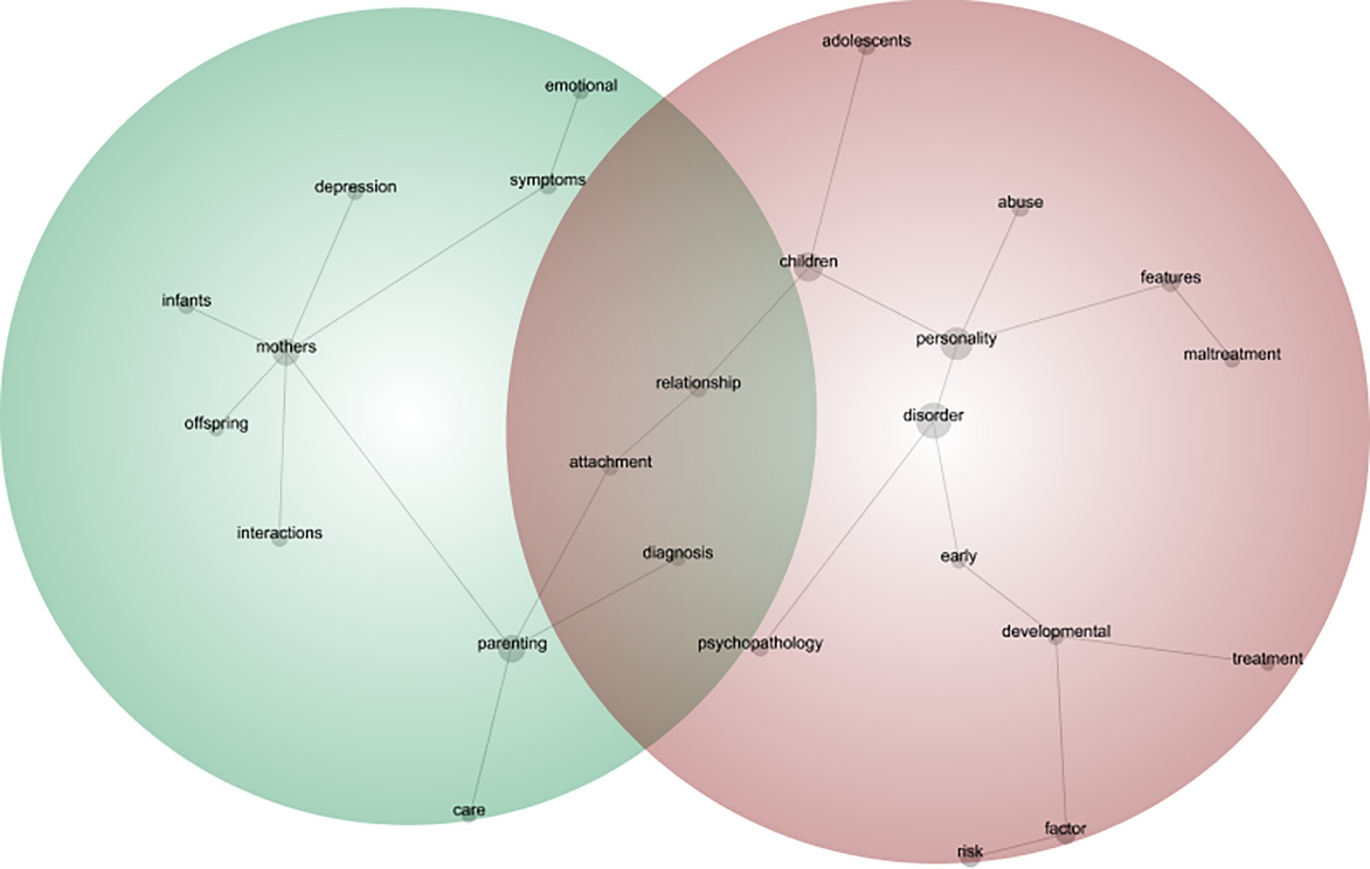
Parenting and personality disorder thematic and concept map.

Using Leximancer, we developed a concept map (Fig 2) to depict visually the relationship between identified themes and concepts across the eight systematic reviews. The concept map identified two distinct themes of ‘parental vulnerability’ and ‘early developmental vulnerability’. The theme of parental vulnerability is comprised of concepts such as ‘mothers’, ‘symptoms’, ‘emotional’, ‘interactions’, ‘infants’, ‘attachment’, ‘depression’, ‘offspring’ and ‘care’, whilst the theme of early developmental vulnerability is comprised of concepts such as ‘risk’, ‘factors’, ‘abuse’, ‘maltreatment’, ‘development’ ‘treatment’, ‘diagnosis’, ‘children’. The relationship between concepts is also indicated by the connectivity of concept dots on the visual map. For example, the relationship between mothers and their offspring (i.e. mother-offspring interactions) is described by five systematic reviews. Notably, the concepts ‘attachment’, ‘relationship’ and ‘diagnosis’ are present in both themes. For a summary of results, see Table 2.

**Table 2:** Summary of key themes and sub-themes for qualitative analysis of systematic reviews.

Key Themes	Early developmental vulnerability	Parental vulnerability
Leximancer Connectivity	100%	53%
Sub-themes (*n*) Examples of related text	Disorder (*n* = 813) E.g. “the majority (n = 9) of the 11 studies included in this review provide evidence to suggest that personality *disorder* amongst others exerts a negative impact on parenting”	Mothers (*n* = 570) E.g. “Eliot et al. (2014) found that *mothers* with BPD scored significantly higher on self-reported overprotection than HCs”
	Personality (*n* = 672) E.g. “Despite increasing attention given to the prognosis, consequences and correlates of *personality* disorder, comparatively little is known about the etiology of these disorders”	Parenting (*n* = 540) E.g. “In two infant studies, mothers with BPD reported significantly higher *parenting* stress and distress”
	Children (*n* = 552) E.g. “In a well documented paper… it was clarified that patients with BPD reported more *childhood* traumas in comparison to other personality disorders”	Symptoms (*n* = 214) E.g. “Professional intervention could be aimed at improving verbal communication between adolescents with BPD *symptoms* and their parents and could therefore contribute to minimise the detrimental effects of verbal abuse on self-esteem”
	Risk (*n* = 280) E.g. “Parental divorce was also associated with higher *risk* of developing borderline features in one study”	Emotional (*n* = 166) E.g. “Findings suggest that vulnerability from mother to offspring may be partly transmitted via maladaptive parenting and maternal *emotional* dysfunction”
	Factor (*n* = 258) E.g. “Failure to develop a secure base and attachment trauma were generally identified as potential *factors* explaining the aetiology of this personality disorder”	Interactions (*n* = 159) E.g. “Mothers with BPD smiled less, touched and imitated their infants less and played fewer games with their babies. Lack of sensitivity in *interactions* with offspring is a recurring theme”
	Relationship (*n* = 187) E.g. “Difficulties relating to other people and developing close and meaningful intimate *relationships* can be seen across all 10 personality disorders, albeit to varying degrees”	Infants (*n* = 133) E.g. “All three personality disorder clusters were found to exert a detrimental main effect on *infant* care practices such that mothers with these disorders were less likely to employ recommend care practices than other mothers”
	Abuse (*n* = 226) E.g. “Compared to participants with Axis-I disorders… BPD participants consistently reported more frequently parental *abuse* and neglect”	Attachment (*n* = 134) E.g. “BPD participants were more likely to have unresolved issues regarding childhood trauma and to have *attachment* issues”
	Adolescents (*n* = 222) E.g. “Indeed, clinical research has demonstrated that significant reductions in borderline symptoms, and associated dysfunction, can be gained through interventions during the *adolescent* years”	Depression (*n* = 143) E.g. “Children of mothers with BPD had poorer mental health than control groups, showing substantially elevated levels of *depression*”
	Features (*n* = 191) E.g. “The 10 studies included in this systematic review used a variety of different methods to assess either borderline personality *features* or BPD”	Offspring (*n* = 114) E.g. “Risk for *offspring* PD increased steadily as a function of the number of problematic parenting behaviours that were evident”
	Developmental (*n* = 133) E.g. “Thus, attachment theory is relevant to the *development* of both personality and parenting styles in adulthood”	Care (*n* = 100) E,g. “More precisely, BPD daughters reported less parental *care*, more maternal overprotection and inconsistent parental values and norms, while their mothers and fathers both described themselves in a more normative fashion”
	Psychopathology (*n* = 131) E.g. “The main aim of the current review was to examine associations between *psychopathologica*l (i.e. Psychiatric disorders ad suicidality) and aetiological (i.e. adverse life events) factors identified a priori in the adult literature and the BPD diagnosis in youth populations”	
	Maltreatment (*n* = 132) E.g. “compared to his or her *non-maltreated* twin, the physically *maltreated* twin exhibited more borderline personality related characteristics”	
	Diagnosis (*n* = 128) E.g. “Excessive separation anxiety as infant was a significant predictor of BPD *diagnosis* across all developmental periods for men only”	
	Early (*n* = 73) E.g. “There is an urgent need to identify signs that harbinger onset of borderline personality disorder (BPD). Advancement in this area is required to refine developmental theories, discover etiological mechanism, improve *early* detection, and achieve our ultimate goal of prevention”	
	Treatment (*n* = 63) E.g. “Emerging evidence indicates that *treatment* gains may be enhanced by interventions that are distinct from these commonly used to *treat* internalising and externalising disorders”	

*Note*: the Leximancer Connectivity percentage indicates the relative importance of each theme (e.g., the higher the percentage, the more important the theme). The percentage is calculated using the connectedness of concepts within that theme.

## Discussion

The present overview systematically reviewed and qualitatively synthesised the research on the relationship between parenting and personality disorder. We identified eight systematic reviews and 140 primary studies (120 unique studies), which met the criteria for inclusion. These studies included a total sample of 121,895 participants, of which 84,333 (69.18%) were uniquely sampled. The majority of primary studies focused on borderline personality pathology (*n* = 100) in community settings (*n* = 102) using a case-control study design (*n* = 62). Methodological quality varied amongst the systematic reviews and results were qualitatively synthesised in all but one [44] systematic review. We found that maladaptive parenting practices were overwhelmingly reported as a psychosocial risk factor for the development of borderline personality disorder pathology [37, 44–47]. Furthermore, borderline personality disorder was found to be associated with maladaptive parenting, and negative offspring and parenting-offspring outcomes [16, 42, 43]. Through visually mapping the eight systematic reviews, two distinct themes of ‘parental vulnerability’ and ‘early developmental vulnerability’ emerged, with dynamic interactional processes related to the parent-offspring relationship (e.g. attachment and care), psychosocial risk factors (e.g. abuse and maltreatment), and personality disorder pathology (e.g. symptoms, treatment and diagnosis). Of interest, the concepts ‘attachment’, ‘relationship’ and ‘diagnosis’ are present in both the themes of parenting and personality disorder. As such, strengthening the parent-child attachment relationship and providing parents with an appropriate personality disorder diagnosis (and therefore evidence-based treatment), may prove to be important target areas for clinical practice and policy.

### Limitations

This study is an overview of existing systematic reviews and as such, relied on the information presented by the included reviews and the relevant primary studies. With this in mind, a few key methodological limitations are considered. We found that the quality of the systematic reviews included in this overview varied. For example, five systematic reviews did not have a clearly defined research question [37, 42, 44–46], and three did not report a quality assessment tool [37, 46, 47]. These methodological limitations may have influenced search strategy, extraction of data, the quality of data reported and the analysis or synthesis of results. Although we thoroughly developed our search strategy according to the PRISMA guidelines and pre-registered our overview with PROSPERO protocol, we may have missed relevant systematic reviews. For example, systematic reviews and primary studies that were published outside of the relevant search dates. Moreover, this overview may also be subject to the ‘file drawer’ problem, whereby the included studies report significant findings only. We tried to circumvent this by investigating the primary studies included in the systematic reviews. However, we did not include a quality assessment or risk of bias for these primary studies and as such, it is possible that the accuracy and generalisability of the findings of the present overview may be compromised.

Another methodological limitation of this overview is that results were drawn from systematic reviews in which there is a relatively high degree of crossover in included primary studies. Forty-two (30%) of the reported primary studies were included in two or more systematic reviews, with 20 of the primary studies drawn from the same sample. For example, the CIC cohort was sampled in eight primary studies [49–56] and three systematic reviews [37, 42, 46]. Although the majority of participants are estimated to be unique, this relatively high degree of crossover between primary studies and systematic reviews may have led to the overstatement of findings, particularly concerning the number of parent-offspring studies drawn from the same sample in systematic reviews exploring the transmission of personality disorder [16, 42, 43]. Unfortunately, it was outside the scope of the current overview to control for this when interpreting the results of the included systematic reviews.

Additionally, many of the systematic reviews included in this overview reported information from primary studies that utilised cross-sectional designs, relied on self-report measures for personality disorder pathology, retrospective self-report measures for maladaptive parenting and did not control for confounding variables, and as such, a causal relationship between maladaptive parenting and personality disorder cannot be determined. Yet we found that a number of systematic reviews reporting on cross-sectional studies also make causal statements. For example, Eyden et al [16] state in their abstract “Findings suggest that vulnerability (for BPD/BPD symptoms) from mother to offspring may be partly transmitted via maladaptive parenting and maternal emotional dysfunction” (p. 85). However, on further investigation this statement appears to be generated from the results of one prospective community-based family cohort study [35] and one cross-sectional study [57], in which maladaptive parenting is defined using heterogeneous constructs. Consequently, we recommend that results inferring causality are interpreted with caution.

There was a significant dearth in studies exploring the complex relationship between individual and environmental process in formation of personality disorder. Only one systematic review [37] considered the role of the child vulnerability factors and maladaptive parenting in the aetiology of BPD, and one [16] identified offspring characteristics as potential mechanisms underpinning the transmission of vulnerability for BPD to mother to offspring. Consequently, this overview synthesised the research on one particular environmental stressor (i.e. maladaptive parenting) implicated in the aetiology of personality disorder, and as such, we are not able to make comment on the role of individual temperament or genetic vulnerability or the interaction between these two variables. Moreover, the vast majority of included systematic reviews (*n* = 7, 87.50%) and primary studies (*n* = 100, 71.43%) reported on borderline personality pathology specifically. Although 40 (28.57%) primary studies included in this overview explored other non-borderline personality disorders (e.g. NPD), these results were reported in one systematic review only [42]. All systematic reviews exploring the impact of personality disorder on parenting also principally reported on the impact of BPD on mothers and mother-offspring interactions. Consequently, the results of this overview also principally relate to association between maladaptive parenting and BPD, and the impact of BPD on mothers, their offspring and the mother-offspring relationship.

### Implications for research

The included systematic reviews predominately focused on BPD, and do not allow us to draw conclusions on the relationship between maladaptive parenting practices and other personality disorders. The lack of information reported on other personality disorders is of concern given research suggesting that personality disorder more generally places parents and their offspring at risk [42], and thus is an important area for future research. Future research is needed to explore the relationship between parenting and other personality disorders, or using a dimensional approach to personality disorders as recommended by the DSM-5 alternate model of personality disorders and ICD-11 [38]. The lack of information about the impact of paternal personality disorder on fathers, offspring and father-offspring interactions is problematic given research suggesting that personality disorder (particularly BPD) occurs equally amongst genders in the general population [5]. Further research using a male population is therefore greatly needed.

Systematic reviews populated by cross-sectional studies do not allow us to infer causality between parenting and personality disorder variables. To confirm the impact of personality disorder on parenting practices, and parent and child outcomes, future studies would benefit from adopting more rigorous designs (e.g. prospective longitudinal studies). Additional epidemiological study designs (such as twin studies), may also provide a stronger argument for causality between variables and enhance our understanding of the complex relationship between child temperament (e.g. hypersensitivity) and environmental stressors (e.g. maladaptive parenting) in the development and expression of personality disorder pathology. Many primary studies reported in the included systematic reviews also relied on self-report measures to determine personality disorder pathology and history of maladaptive parenting. To reduce the risk of bias, future studies would benefit from utilising a combination of self, other and observational methods. Missing information uncovered by quality assessment (e.g. lack of research aims or quality assessment) is also of concern given that systematic reviews are intended to be a high quality study design for analysing, synthesising and translating research. Future systematic reviews would therefore benefit from adopting a standardised protocol such as the PRISMA guidelines [39] and ensuring that they include an adequate quality assessment. Furthermore, the majority of studies (*n* = 7) included in this overview qualitatively synthesised their results, and as such, future studies would benefit from adopting a quantitative or mixed methods approach.

### Implications for clinical practice and policy

The present overview consolidates evidence for the association between maladaptive parenting, personality disorder and parent and offspring outcomes. Our findings suggest that to work more effectively with parents with BPD and help break the cycle of intergenerational complex mental health issues, standard treatment for BPD should create space for individuals to explore difficulties with parenting, including how they were parented and their early attachment relationships. When working with parents with BPD, treatment providers and their clients may benefit from spending time looking at the individual’s early experiences of how they were parented and drawing their attention to how these early attachment relationships and subsequent learning experiences may be affecting the way they parent with their own children.

There is also a significant dearth in parenting interventions specifically developed for this population (for preliminary work see [58–60]). This is concerning given the findings of the present overview, and may highlight a divide between psychological research and clinical practice. To ensure that people with personality disorder and their families are receiving appropriate care, it essential that we continue to translate current research into clinical practice and policy. Moreover, research investigating the intergenerational transmission of complex mental health issues (such as personality disorder) is still in its relative infancy. In order build on this evidence base, future studies would benefit from longitudinally following the offspring of parents with personality disorder from the antenatal period throughout the lifespan, and investigating the underlying mechanisms underpinning the relationship between maladaptive parenting and personality disorder.

## Conclusion

Individuals with borderline personality pathology retrospectively recall maladaptive parenting in their childhood at a rate significantly higher than psychiatric and healthy comparisons or controls. Consequently, maladaptive parenting is hypothesised to be prospectively associated with the development of borderline personality pathology (including diagnosis, symptoms and features). Maladaptive parenting practices are present in some individuals with personality disorder, and are associated with negative parental, offspring and parent-offspring relationship outcomes. These findings have led authors to suggest that maladaptive parenting may be a potential mediating factor in the intergenerational transmission of BPD. However, these conclusions are based on a body of evidence that varies in methodological design and quality, and as such, future research utilising more rigorous methodology, such as longitudinal designs, is needed. Additionally, further work incorporating epigenetic processes hold promise to enhance our understanding of the complex relationship between individual and environmental processes in the development and expression of personality disorder pathology. In order to break the cycle of complex mental health issues, a greater emphasis should be placed on parenting in clinical practice and parenting interventions need to be specifically designed and empirically tested for this population. These interventions would likely benefit from first ensuring that parents receive an appropriate diagnosis, before focusing on enhancing the parent-child attachment relationship.

## Supporting information

S1 Checklist PRISMA checklist. (DOC)

S1 Table Systematic review checklist for quality assessment. (DOCX)

S2 Table Primary studies with adult participants included in systematic reviews. (DOCX)

S3 Table Primary studies with child and adolescent participants included in systematic reviews. (DOCX)

S4 Table Primary studies with parent-offspring participants included in systematic reviews. (DOCX)

S1 Text Acronym key. (DOCX)
