# Supplementary material for: Parenting and personality disorder: An overview and meta-synthesis of systematic reviews
Source: PLoS One. 2019 Oct 1;14(10):e0223038. doi: 10.1371/journal.pone.0223038 (PMC6772038; doi:10.1371/journal.pone.0223038)
Supplement: S1 Table — (DOCX) [file pone.0223038.s002.docx]

|  |  | Keinanen et al. (2012) | Laulik et al.  (2013) | Petfield et al.  (2015). | Eyden et al. (2016) | Stepp et al.  (2016) | Winsper et al.  (2016) | Boucher et al. (2017) | Ibrahim et al.  (2018) |
| --- | --- | --- | --- | --- | --- | --- | --- | --- | --- |
| Section A: Are the results of the review valid? | 1. Did the review address a clearly focused question? | -  The reviewers address the purpose of the review without clearly defining the research questions | -  The reviewers address the purpose and aims of the review without clearly defining the research question. | Yes | Yes | -  The reviewers address the purpose and goals of the review without clearly defining the research questions. | -  The reviewers address the aims of the review without clearly defining the research questions. | Yes | -  The reviewers address the purpose of the review without clearly defining the research question. |
|  | 1. Did the authors look for the right type of papers? | Yes | Yes | Yes | Yes | Yes | Yes | Yes | Yes |
|  | 1. Do you think all the important, relevant studies were included? | -  Searches were conducted in only two databases (PsycINFO and MEDLINE). However, these are major databases with a wide scope. The reviewers did not conduct hand searches. | Yes | -  Searches were conducted in only two databases (PsycINFO and MEDLINE). However, these are major databases with a wide scope. | Yes | Yes | Yes | -  The reviewers used appropriate databases but did not conduct hand searches. Additionally, the authors note that stringent inclusion/exclusion criteria may have resulted in eligible studies not being included. | Yes |
|  | 1. Did the reviews authors do enough to assess quality of the included studies? | -  No quality assessment reported | Yes | Yes | Yes | -  No quality assessment reported | Yes | -  No quality assessment reported | Yes |
|  | 1. If the results of the review have been combined, was it reasonable to do so? | Yes | Yes | Yes | Yes | Yes | Yes | Yes | Yes |
| Section B: What are the results? | 1. What are the overall results of the review? | 51 papers were retrieved to evaluate the risk factors for the aetiology of BPD that meet the interventional evidence-based medicine (EBM) criteria of best evidence. Five vulnerability factors were identified and classified according to the EBM criteria:   1. BPD risk factor 1: Childhood trauma/abuse 2. BPD risk factor 2: Unfavourable parenting 3. BPD risk factor 3: Object relations 4. BPD risk factor 4: Insecure attachment/loss 5. BPD risk factor 5: Symbolisation-reflectiveness capacity | Nine out of 11 studies included for review found evidence to support the existence of a positive association between a diagnosis of personality disorder and personality disorder features and impaired parenting behaviours after controlling for confounding factors. In these studies, the presence of personality disorder was related to:   - The use of inadvisable and problematic parental practices - Inconsistent parental discipline - Low parental affection, assistance, praise and encouragement - Less satisfaction and reported competence in the parenting role - Sensitive, instructive poorly attuned and disrupted parent-infant interactions - Harsh behaviour - Frightening/disoriented parental behaviour - Status as an abusive parent. | - Mother’s BPD diagnosis was associated with differences in parenting outcomes compared to control group, including: reduced sensitivity and increased intrusivity towards child; difficulty un structured activities and having poorer levels of family organisation; family environments characterised by high levels of hostility and low levels of cohesion; increased overprotection; poor mind-mindedness; less competence and satisfaction in parenting role; and increased parenting stress. - Mother’s BPD diagnosis was also associated with differences in children’s outcomes compared to control groups, including: less satisfying interactions (e.g., more looking away and dazed looks); more cognitive-behavioural risk factors (e.g., poorer theory of mind); difficulties in mother-child relationship (e.g., disrupted attachment style) and poorer mental health (e.g., depression). | - Mothers with BPD/BPD symptoms appear less sensitive, more intrusive, more overprotective, and more hostile, show less engagement, and are more likely to have maladaptive interactions with their offspring compared to controls. - Offspring exhibited a range of psychological and psychosocial outcomes across several stages of development, including BPD symptoms/features. - Potential mechanisms underpinning the transmission of vulnerability from mother to offspring include: maladaptive parenting, maternal emotional dysfunction and offspring characteristics. | Multiple factors across social, familial, maltreatment, and child domains increase the risk for BPD. The most robust risk indicators in there domains were:   - Social: low SES, stressful life events, family adversity - Family: maternal psychopathology, affective parenting dimension (low warmth, hostility, harsh punishment) - Maltreatment: physical or sexual abuse, neglect - Child: low IQ, negative affectivity and impulsivity, internalising and externalising psychopathology | - Youth BPD was found to share a number of features with the adulthood disorder. - The common aetiological features were:  1. Sexual and physical abuse 2. Maladaptive parenting 3. Neglect 4. Parental conflict  - The common psychopathological features were:  1. Comorbidity with other psychiatric disorders (e.g., mood disorders, anxiety disorders, substance abuse, eating disorder, PTSD) 2. Suicide (attempt and ideation) and self-harm | - BPD participants and their parents consistently reported a much more dysfunctional PCR compared to normal and clinical controls. - Parental care and overprotection consistently discriminated BPD from NC participants. However, these two variables are related to severity of psychology, rather than being a risk factor of BPD. - Relational adversity does not sufficiently explain BPD development. Parental inconsistency may more a more appropriate risk factor for the aetiology of BPD. | - Four of 10 studies found that children with BPD/borderline features were more likely to have a history of maltreatment compared to those with other clinical presentations - Six studies showed that maltreated children compared to non-maltreated child were more likely to present with borderline features. - There was some evidence that all types of abuse and neglect were independently associated with borderline features. - For children who had experienced more than one type of abuse, there was evidence of a cumulative effect of maltreatment, resulting in those who has experienced maltreatment across more development periods showing significantly higher levels of borderline features. - These findings suggest that maltreatment in general is a risk factor for borderline features in children and BPD in adults, with more severe abuse increasing the risk of developing borderline features. |
|  | 1. How precise are the results? | -  Data was synthesised using a qualitative approach | -  Data was synthesised using a qualitative approach | -  Data was synthesised using a qualitative approach | -  Data was synthesised using a qualitative approach | -  Data was synthesised using a qualitative approach | Statistically significant pooled associations for adult and youth BPD were observed for sexual abuse, physical abuse, maternal hostility/verbal abuse and neglect. Several adult psychopathological features were also associated with youth BPD, including comorbid mood, anxiety, substance use disorders, self-harm, suicide ideation and suicide attempt. However, not all relevant psychopathological (e.g., neurobiological) and aetiological (e.g., biological predisposition), insecure attachment factors could not be quantitatively synthesised. | -  Data was synthesised using a qualitative approach | -  Data was synthesised using a qualitative approach |
| Section C: Will the results help locally? | 1. Can the results be applied to the local population? | Yes | Yes | Yes | Yes | Yes | Yes | Yes | Yes |
|  | 1. Were all important outcomes considered? | Yes | Yes | Yes | Yes | Yes | Yes | -  The authors note that stringent inclusion/exclusion criteria may have resulted in eligible studies not being included. | Yes |
|  | 1. Are the benefits worth the harm and costs? | Yes | Yes | Yes | Yes | Yes | Yes | Yes | Yes |
