# Supplementary material for: Parenting and personality disorder: An overview and meta-synthesis of systematic reviews
Source: PLoS One. 2019 Oct 1;14(10):e0223038. doi: 10.1371/journal.pone.0223038 (PMC6772038; doi:10.1371/journal.pone.0223038)
Supplement: S2 Table — (DOCX) [file pone.0223038.s003.docx]

| Primary studies included in systematic reviews | | | | | | | | Systematic reviews | | | | | | | |
| --- | --- | --- | --- | --- | --- | --- | --- | --- | --- | --- | --- | --- | --- | --- | --- |
| Author (date) Country | PD construct: assessment (cut-point) | Setting | Design | N  (clinical group/s: n) | Gender  % female | Age  M ± SD: range  years | Race  % Caucasian | Keinanen et al. (2012) | Laulik et al. (2013) | Petfield et al. (2015) | Eyden et al. (2016) | Stepp et al. (2016) | Winsper et al. (2016) | Boucher et al. (2017) | Ibrahim et al. (2018) |
| Aaronson et al. (2006)  USA | BPD dx, OCPD dx: DIPD-IV | Clinical: IN | Longitudinal Cohort (CLPS) | 90  (BPD: 50, OCPD: 40) | 82% (BPD: 90%, OCPD: 72.5%) | 31.7 ± 8.3: 21-50 | 66% | ✓ |  |  |  |  |  |  |  |
| Allen et al. (2005)  USA | BPD dx, ST-BPD dx: SCID-II | Clinical: OUT | Case-control | 93  (BPD: 40, ST-BPD: 10, PC: 24, HC: 19) | 69.5% | 34: 23-48 | 58.9% |  |  |  |  |  |  | ✓ |  |
| Baker et al. (1992)  USA | BPD dx: DIB (≥7) | Clinical: IN, Community: POP | Cross-sectional | 60  (BPD: 31, MDD: 15, HC: 14) | BPD: 87.1%, MDD: 60.0%, HC: 64.3% | BPD: 30.4 ± 8,  MDD: 40.7 ± 9.4,  HC: 37.8 ± 11.1 | N/R |  |  |  |  |  |  | ✓ |  |
| Bandelow et al. (2005)  Germany | BPD dx: SCID-II | Clinical: OUT, Community: POP | Case-control | 175  (BPD: 66, HC: 109) | BPD: 71.2%, HC: 60.6% | BPD: 30.2 ± 9.4,  NC: 32.3 ± 6.6 | N/R | ✓ |  |  |  |  |  | ✓ |  |
| Barone (2003)  Italy | BPD dx: SCID-II | Clinical: OUT, Community: POP | Case-control | 80  (BPD: 40, HC: 40) | BPD: 62.5% | BPD: 29 ± 6.3 | N/R | ✓ |  |  |  |  |  |  |  |
| Bierer et al. (2003)  USA | PD dx: SCID-II | Clinical: OUT | Cross-sectional | 182  (CA: 92, CB: 94, CC: 115) | 35.16% | 37.8 ± 8.4 | N/R | ✓ |  |  |  |  |  |  |  |
| Bradley (2005)  USA | BPD sxs: SWAP-200 | N/R | Cross-sectional | 524 (clinicians reporting on 524 BPD patients) | 47% | 39 ± 11.5 | N/R | ✓ |  |  |  |  |  |  |  |
| Byrne et al. (1990)  Canada | BPD dx: DSM-III criteria-based dx | Clinical: IN + OUT | Cross-sectional | 29  (BPD: 15, SZ: 14) | BPD: 86.7%, SZ: 71.4% | BPD: 24.6, SZ: 25.9 ± 3.3 | N/R |  |  |  |  |  |  | ✓ |  |
| Carlson, Egeland & Srouffe (2009)  USA | BPD sxs: SCID-II, SCID-NP | Community: HR | Longitudinal cohort (Egeland, 1991) | 162  (N/R) | 49% | 20.7 ± 3.6: 14-34 | 67% |  |  |  |  | ✓ |  |  |  |
| Cohen et al. (2008)  USA | BPD sxs: CIC-SR | Community: POP | Longitudinal cohort (CIC) | 680 (also reports 787)  (N/R) | N/R | 33.2 ± 2.9 | 91% |  |  |  |  | ✓ |  |  |  |
| Crawford et al. (2009)  USA | BPD sxs: CIC-SR | Community: POP | Longitudinal cohort (CIC) | 766 (also reports 678)  (N/R) | N/R | 33.1 ± 2.8 | 91% |  |  |  |  | ✓ |  |  |  |
| Dubo et al. (1997)  USA | BPD dx, BPD sxs (SH): DIB-R, DIPD-R, LBSI, MSI-BPD | Clinical: IN | Case-control (McLean hospital) | 59  (BPD: 42, OPD: 17) | BPD: 71.4%, OPD: 58.8% | BPD: 31.7, OPD: 35.9: 18-60 | N/R |  |  |  |  |  |  | ✓ |  |
| Famularo, Kinscherff & Fenton (1992)  USA | PD dx: SCID-II | Community: HR | Case-control | 91  (MP: 54, NMP: 37) | 100% | MP: 35.4, NMP: 32.1 | MP: 43%  NMP: 41% |  | ✓ |  |  |  |  |  |  |
| Fletcher et al. (2014)  Australia | BPD dx: DIPD-IV, SCID-II | Clinical: OUT, Community: POP | Cross-sectional | 48  (BPD: 24, BP-II: 24) | BPD: 87.5%, BP-II: 50% | 32.9 ± 11.3 (BPD: 36.7, BP-II: 32.8) | N/R |  |  |  |  |  |  | ✓ |  |
| Fonagy et al. (1996)  UK | PD dx: SCID-II | Clinical: IN + OUT | Case-control | 167  (Axis I + II: 82, PC: 85) | Axis I + II: 81.7%,  PC: 82.4% | 29 | N/R | ✓ |  |  |  |  |  |  |  |
| Fossati et al. (2001)  Italy | BPD dx, CB dx, CAC dx: SCID-II | Clinical: IN + OUT, Community: POP | Case-control | 457  (BPD: 44, CB: 98, CAC: 39, PC: 70, HC: 206) | BPD: 56.8%, CB: 54.1%, CAC: 64.1%, PC: 75.7%, HC: 82.0% | BPD: 27.6 ± 7.2,  CB: 30.4 ± 8.7,  CAC: 31 ± 8.6,  PC: 30.8 ± 8.7,  HC: 24.4 ± 8.8 | N/R |  |  |  |  |  |  | ✓ |  |
| Frank & Hoffman (1986)  Canada | BPD dx: DIB-2 (≥7) | Clinical: OUT | Cross-sectional | 24  (BPD: 10, NEU: 14) | 100% | BPD: 27.4, NEU: 25.1: 21-33 | N/R |  |  |  |  |  |  | ✓ |  |
| Ghiassi et al. (1986)  Germany | BPD dx: SCID-II | Clinical: IN, Community: POP | Case-control | 70  (BPD: 50, HC: 20) | BPD: 92%, HC: 65% | BPD: 26.2 ± 4.2,  HC: 26 ± 4.2; 18-41 | N/R |  |  |  |  |  |  | ✓ |  |
| Goldberg et al. (1985)  USA | BPD dx: DSM-III dx criteria-based dx | Clinical: IN, Community: HP | Case-control | 56  (BPD: 24, PC: 22, HC: 10) | N/R | BPD: 24.9 ± 6.0,  PC: 23.4 ± 12.7,  HC: 28.4 ± 8.4 | N/R |  |  |  |  |  |  | ✓ |  |
| Hayashi et al. (1995)  Japan | BPD dx: DSM-III R criteria based dx | Clinical: IN | Case-control | 26  (BPD: 13, PC: 13) | BPD: 77%,  PC: 85% | BPD: 27 ± 6.4,  PC: 30 ± 8.4 | H/R |  |  |  |  |  |  | ✓ |  |
| Heffernan & Cloitre (2000)  USA | BPD + PTSD dx: SCID-II | Community: POP | Cross-sectional | 71  (BPD + PTSD: 26, PTSD: 45) | 100% | 37.3 ± 11 | 63% |  |  |  |  |  |  | ✓ |  |
| Helgeland & Torgersen (1997)  Norway | BPD dx: SCID-II | Clinical: IN, Community: UND | Cross-sectional | 48  (BPD: 14, SZ: 19, HC: 15) | BPD: 78.6%, SZ: 26.3%, HC: 46.7% | BPD: 30.9, SZ: 27.9, HC: 26 | N/R |  |  |  |  |  |  | ✓ |  |
| Herman et al., (1989)  USA | BPD dx, SZ-PD dx, ASPD dx: DSM-III (≥5), BPS (≥150) | Clinical: OUT | Longitudinal cohort (Perry 1985) | 55  (BPD: 21, BPT: 11, BP-II: 11, ASPD: 6, SZ-PD: 6) | BPD: 81%,  BPT: 0%, BP-II: 54.5%, ASPD: 50%, STPD: 50% | N/R | N/R | ✓ |  |  |  |  |  |  |  |
| Hernandez et al (2012)  Spain | BPD dx, OPD dx: SCID-II, DIB-R | Clinical: IN + OUT | Case-control | 109  (BPD: 32, OPD: 43, PC: 34) | 100% | 38.8 ± 10.7: 19-64 | N/R |  |  |  |  |  |  | ✓ |  |
| Huang et al. (2014)  China | BPD dx, OPD dx: MSI-BPD (≥7), SCID-II | Clinical: OUT, Community: POP | Cross-sectional | 286  (BPD: 152, OPD: 79, PC: 55) | 59.1%  (BPD: 67.1%, OPD: 51.9%, PC: 47.3%) | 28.4 ± 9.1 | N/R |  |  |  |  |  |  | ✓ |  |
| Joyce et al. (2003)  USA | BPD dx, AVPD dx: SCID-II, SCID-PQ | Clinical: OUT | Longitudinal cohort (Joyce at al., 2002) | 73  (BPD: 30, AVPD: 43) | 58.90% | 18-35 | N/R |  |  |  |  |  |  | ✓ |  |
| Katerndahl (2005)  USA | BPD dx: SCID-II | Clinical: OUT | Cross-sectional (CEAS) | 100  (BPD: 92) | 100% | 28.7 ± 7.0: 18-40 | N/R (69% Hispanic) |  |  |  |  |  |  | ✓ |  |
| Krabbendam et al. (2015)  Netherlands | BPD dx. ASPD dx: SCID-II | Clinical: INC | Longitudinal cohort | 184  (BPD: 17, ASPD: 29, ASPD+BPD: 28, PC: 110) | 100% | 20 ± 1.4: 16.2-24.1 | N/R (57.6% Dutch, 25.9% non-Western, 6.5% other Western) |  |  |  |  | ✓ |  |  |  |
| Laporte & Guttman (1996)  Canada | BPD dx, OPD dx: N/R (“clinical evaluation”) | Clinical: OUT | Case-control | 751  (BPD: 366, OPD: 385) | 100% | 16-45 | N/R | ✓ |  |  |  |  |  |  |  |
| Laporte & Guttman (2007)  Canada | BPD dx: DIB-R | Clinical: OUT, Community: Sisters | Case-control | 102  (BPD: 35, AN: 34, HC: 33) | 100% | BPD: 31.7 ± 6,  AN: 23.5 ± 7,  HC: 23.4 ± 7: 16-40 | N/R |  |  |  |  |  |  | ✓ |  |
| Laporte et al. (2011)  Canada | BPD dx: DIB-R (8/10), DIPD-IV, DSM-IV-TR criteria based dx | Clinical: OUT, Community: Sisters | Case-control | 112 ((BPD-HC (sister dyads): 56)) | 100% | BPD: 28.7, HC: 30.2: 18-45 | N/R |  |  |  |  |  |  | ✓ |  |
| Laporte et al. (2012)  Canada | BPD dx: DIB-R, DIPD-IV, DSM-IV-TR criteria based dx | Clinical: OUT, Community: Sisters | Case-control | 106 ((BPD-HC (sister dyads): 53)) | 100% | BPD: 28.2 ± 7.3,  HC: 29.8 ± 7.6: 18-45 | N/R |  |  |  |  |  |  | ✓ |  |
| Lobbestael et al. (2006)  Netherlands | BPD dx, APD dx: SCID-II | Clinical: IN, OUT + INC, Community: HP | Case-control | 48  (BPD: 16, APD: 16. HC: 16) | 50% | 30.9 (BPD: 31.4, APD: 31.1, HC: 30.2) | N/R | ✓ |  |  |  |  |  |  |  |
| Machizawa-Summers (2007)  Japan | BPD dx: DSM-IV-TR criteria based dx, BSI (≥28) | Clinical: OUT | Case-control | 90  (BPD: 45, PC: 45) | 100% | BPD: 28.5 ± 6.47,  PC: 32.9 ± 7.62 | N/R |  |  |  |  |  |  | ✓ |  |
| Merza et al. (2015)  Hungary | BPD dx: SCID-II | Clinical: IN, Community: POP | Case-control | 204  (BPD: 80, MDD: 73, HC: 51) | BPD: 85%; MDD: 82.2%; HC: 86.3% | BPD: 30.5 ± 10.9, MDD: 44.3 ± 5.9, HC: 33.6 ± 8.7 | N/R |  |  |  |  |  |  | ✓ |  |
| Minzenberg et al. (2006)  USA | BPD dx: SCID-II | Clinical: OUT, Community: POP | Case-control | 67  (BPD: 41, HC: 26) | BPD: 88%; HC: 89% | BPD: 35.3 ± 12.9,  HC: 34.4 ± 9.3 | BPD: 76%,  HC: 77% | ✓ |  |  |  |  |  |  |  |
| Paris et al. (1993)  Canada | BPD-L, BPD-A: DIB-R | Clinical: OUT | Longitudinal cohort (Paris et al., 1994a) | 39  (BPD-L: 13, BPD-A: 26) | 100% | BPD-L: 34.9,  BPD-A: 37.7 | N/R | ✓ |  |  |  |  |  | ✓ |  |
| Paris et al (1994)  Canada | BPD dx, OPD dx: DIB-R | Clinical: OUT | Longitudinal cohort (Paris et al., 1994a) | 150  (BPD: 78, OPD: 72) | 100% | BPD: 28.3 ± 6.3,  OPD: 29.7 ± 7.2 | N/R | ✓ |  |  |  |  |  | ✓ |  |
| Paris et al. (1994)  Canda | BPD dx, OPD dx: DIB-R (8/10) | Clinical: OUT | Longitudinal cohort (Paris 1994a) | 121  (BPD: 61, OPD: 60) | 0% | BPD: 30.6 ± 7.9,  OPD: 32.3 ± 6.5 | N/R | ✓ |  |  |  |  |  | ✓ |  |
| Reich & Zanarini (2001)  USA | BPD dx, OPD dx: DIB-R, DIPD-R | Clinical: IN | Case-control (McLean hospital) | 362  (BPD: 290, OPD: 72) | BPD: 80.3%; OPD: 63.9% | BPD: 26.9 ± 5.8,  OPD: 27.0 ± 8.0: 18-35 | BPD: 87% OPD: 86% | ✓ |  |  |  |  |  |  |  |
| Sansone et al. (2005)  USA | BPD dx: N/R | Clinical: IN | Case-control | 97  (BPD: 31, PC: 66) | 59% | 34 ± 10.7: 15-59 | 72% | ✓ |  |  |  |  |  |  |  |
| Schwarze et al. (2015)  Germany | BPD dx: SCID-II | Clinical: IN + OUT, Community: POP | Case-control | 200  (BPD: 100, HC: 100) | BPD: 90%, HC: 90% | BPD: 31.63 ± 9.73,  HC: 32.02 ± 10.25 | N/R |  |  |  |  |  |  | ✓ |  |
| Thatcher, Cornelius & Clark (2005)  USA | BPD sxs: SCID-II | Clinical: IN, OUT, INC, Community: POP | Longitudinal cohort (PAARC) | 524  (AUD: 355, REF: 169) | AUD: 38.6%; REF: 55% | AUD: 22 ± 2.4,  REF: 22 ± 2.4 | AUD: 85.6% REF: 84% |  |  |  |  | ✓ |  |  |  |
| Timmerman & Emmelkamp (2005)  Netherlands | BPD dx: PDQ-R (for PR and HC), IPDE (for FPI), DSM-II-R interview, ICD-10 | Clinical: FPI, INC, Community: POP | Case-control | 500  (BPD FPI: 8, FPI: 39, BPD PR: 43, PR: 192, BPD: 22, HC: 195) | 0% | FPI: 35 ± 11.3,  PR: 33.8 ± 8.9,  HC: 43.6 ± 11.7: 20-71 | N/R |  |  |  |  |  |  | ✓ |  |
| Torgersen & Alnaes (1992)  Norway | BPD dx, OPD dx: SIPD | Clinical: OUT | Case-control | 272  (SZ: 19, BPD: 36, OPD: 165, HC: 52) | BPD: 63%, SZ: 68% | 18-59 | N/R | ✓ |  |  |  |  |  | ✓ |  |
| Trull (2000)  USA | BPDF: PAI-BOR (>38), SIDP-IV, DIB-R, MMPI-BPD | Community: UND | Cross-sectional | 65  (BF+: 21, BF-: 44) | BF+: 57.1%; BF-: 50% | N/R | N/R | ✓ |  |  |  |  |  |  |  |
| Widom et al. (2009)  USA | BPD dx: DIPD-R | Community: HR + POP | Longitudinal cohort (Widom, 1989a) | 896  (MC: 500, NMC: 396) | 51% | 39.5: 30-47 | 60.8% |  |  |  |  | ✓ |  |  |  |
| Wiehe (2003)  USA | NPD characteristics: NPI, HSNS | Community: HR | Cross-sectional | 152  (MP: 52, NMP: 101) | MP: 79%; NMP: 84% | MP: 34 ± 9.5,  NMP: 42 ± 12 | MP: 48%  NMP: 50% |  | ✓ |  |  |  |  |  |  |
| Yen et al. (2002)  USA | BPD dx, STPD dx, AVPD dx, OCPD dx: DIPD-IV | Clinical: OUT | Longitudinal cohort (CLPS) | 653 (STPD: 86, BPD: 167, AVPD: 153, OCPD: 153, MDD: 94) | STPD: 45.3%, BPD: 73.7%, AVPD: 65.4%, OCPD: 59.5%, MDD: 59.6% | STPD: 34.7 ± 7.8,  BPD: 32 ± 7.9,  AVPD: 32.7 ± 8.1, OCPD: 32.7 ± 8.4,  MDD: 32.9 ± 7.9 | STPD: 72.1%, BPD: 71.3%, AVPD: 83%, OCPD: 79.1%, MDD: 75.5% | ✓ |  |  |  |  |  |  |  |
| Zanarni et al. (1989)  USA | BPD dx, ASPD dx, D-OPD dx: SCID-II, DIB, DIPD | Clinical: OUT | Case-control | 105  (BPD: 50, ASPD: 29, D-OPD: 26) | BPD: 66%; ASPD: 27.6%,  D-OPD: 84.6% | BPD: 29.2 ± 6.4,  ASPD: 25.2 ± 6.1, D-  OPD: 31.3 ± 7.2 | BPD: 98%  APD: 96.6%  D-OPD: 84.6% |  |  |  |  |  |  | ✓ |  |
| Zanarini et al. (1997)  USA | BPD dx, OPD dx: DIB-R, DIPD-R | Clinical: IN | Case-control (McLean hospital) | 467  (BPD: 358, OPD: 109) | BPD: 77.1%; OPD: 56.0% | BPD: 27.6 ± 6.8,  OPD: 29.3 ± 9.1 | 89% | ✓ |  |  |  |  |  | ✓ |  |
| Zanarini et al. (2000)  USA | BPD dx, Axis II dx: DIB-R, DIPD-R | Clinical: IN | Case-control (McLean hospital) | 362  (BPD: 290, Axis II: 72) | BPD: 80.3%; Axis II: 63.95% | BPD: 26.9 ± 5.8,  Axis II: 27.0 ± 8.0 | BPD: 87%  Axis II: 86% | ✓ |  |  |  |  |  | ✓ |  |
| Zanarini et al. (2000)  USA | BPD dx, OPD dx: DIB-R, DIPB-R | Clinical: IN | Case-control (McLean hospital) | 467  (BPD: 358, OPD: 109) | BPD: 77.1%, OPD: 56.0% | BPD: 27.6 ± 6.8,  OPD: 29.3 ± 9.1 | 89% | ✓ |  |  |  |  |  |  |  |
| Zanarini et al. (2002)  USA | BPD dx: DIB-R, DIPD-R | Clinical: IN | Cross-sectional (McLean hospital) | 290  (BPD: 290) | 80.30% | 26.9 ± 5.8 | 87% | ✓ |  |  |  |  |  |  |  |
| Zweig-Frank & Paris (1991)  Canada | BPD dx: DIB-R | Clinical: OUT | Case-control | 161  (BPD: 62, PC: 99) | BPD: 54.8%, PC: 54.5% | BPD: 29.6 ± 9.6,  PC: 29.3 ± 8.6 | N/R |  |  |  |  |  |  | ✓ |  |
| Zweig-Frank & Paris (2002)  Canada | BPD: DIB-R | Clinical: IN + OUT | Longitudinal cohort (Paris 1987) | 59  (BPD: 59) | 83% | 50.9 ± 7.6 | N/R |  |  |  |  |  |  | ✓ |  |
| Total (*n* = 58) | | | | 14,167 (BPD: 3909) | 73% | 31.9 7.1 (18.9-45.9) years | 79% |  | | | | | | | |
