# Supplementary material for: Parenting and personality disorder: An overview and meta-synthesis of systematic reviews
Source: PLoS One. 2019 Oct 1;14(10):e0223038. doi: 10.1371/journal.pone.0223038 (PMC6772038; doi:10.1371/journal.pone.0223038)
Supplement: S3 Table — (DOCX) [file pone.0223038.s004.docx]

| Primary studies included in systematic reviews | | | | | | | | Systematic Reviews | | | | | | | |
| --- | --- | --- | --- | --- | --- | --- | --- | --- | --- | --- | --- | --- | --- | --- | --- |
| Author (date) Country | PD construct: assessment (cut-point) | Setting | Design | N  (clinical group/s: n) | Gender  % female | Age  M ± SD: range  years | Race  % Caucasian | Keinanen et al. (2012) | Laulik et al. (2013) | Petfield et al. (2015) | Eyden et al. (2016) | Stepp et al. (2016) | Winsper et al. (2016) | Boucher et al. (2017) | Ibrahim et al. (2018) |
| Arens et al. (2011)  Germany | BPD dx: SCID-II | Community: POP | Longitudinal cohort (SHIP) | 68  (BPD: 17, DEP: 17, HC: 34) | BPD: 82.4%,  DEP: 82.4%,  HC: 82.4% | BPD: 19.7 ± 1.8,  DEP: 20.2 ± 2.3,  HC: 19.4 ± 1.9 | N/R |  |  |  |  |  |  | ✓ |  |
| Atlas (1995)  USA | BPD dx: DIB-R (≥6) | Clinical: IN | Cross-sectional | 38  (PA/SA hx: 26, No PA/SA hx: 12) | 100% | 14.8 ± 1.3: 12.6-17 | N/R |  |  |  |  |  | ✓ |  |  |
| Atlas (1996)  USA | BPD dx: DIB-R (≥6) | Clinical: IN | Cross-sectional | 38  (BPD: 26, PC: 12) | 100% | 14.8 ± 1.3: 12.6-17 | N/R |  |  |  |  |  | ✓ |  |  |
| Belskey et al. (2012)  UK | BPRC: SWAP-200-A | Community: POP | Longitudinal cohort (E-Risk) | 2232 (N/R) | 51% | 5-12 | N/R |  |  |  |  | ✓ | ✓ |  | ✓ |
| Bentivegna (1985)  USA | BPD dx DSM-III criteria-based dx | Clinical: IN + OUT | Case-control | 164  (BPD: 70, PC: 70, IS: 24) | N/R | BPD: 10,  PC: 10.3,  IS: 8.7: 9-10 | N/R |  |  |  |  |  | ✓ |  |  |
| Berzirganian et al. (1993)  USA | BPD dx, NPD dx, HPD dx, CB dx, CAC dx: T3: modified questions of the SCID-II | Community: POP | Longitudinal cohort | T3: 588  (BPD: 57, NPD: 66, HPD: 41, CD: 70, CB: 113, CAC: 118, Axis I: 123) | T3: 48% | T3: 16.4 ± 2.8 | 90% | ✓ |  |  |  | ✓ |  | ✓ |  |
| Biskin et al. (2011)  Canada | BPD dx: R-DIB (≥ 6) | Clinical: OUT | Longitudinal cohort | 47  (N/R) | 100% | 15.1 | N/R |  |  |  |  |  | ✓ |  |  |
| Cichetti et al. (2014)  USA | BPFS: BPFS-C | Community: HR | Case-control (LI SC) | 1051  (MC: 562, NMC: 489) | 50.2% | 10.4 ± 1.3: 8-12 | 10.4% |  |  |  |  |  |  |  | ✓ |
| Goldman et al. (1992)  USA | BPD dx: DSM-III-R dx criteria based dx (≥4), DIB-R | Clinical: OUT | Case-control | 144  (BPD: 44, PC: 100) | 27% | BPD: 10.8 ± 36,  PC: 10 ± 4.3 | N/R |  |  |  |  |  | ✓ |  | ✓ |
| Goodman (2011)  USA | BPD + MDD dx: DIB-R | Clinical: IN, Community: POP | Case-control | 25  (BPD + MDD: 12, HC: 13) | 77% | 15.8 ± 1.1 | N/R |  |  |  |  |  | ✓ |  |  |
| Gratz et al. (2011)  USA | BPF: CPNI (BP subscale) | Community: POP | Cross-sectional | 225  (N/R) | 45% | 12.2 ± .8: 11-14 | 50% |  |  |  |  |  | ✓ |  | ✓ |
| Greenfield et al. (2015)  Canada | BPD sxs: Ab-DIB | Clinical: IN | Longitudinal cohort (Greenfield 2002) | 204  (N/R) | 68.9% | 14.6 ± 1.5 | 69.5% |  |  |  |  | ✓ |  |  |  |
| Greenman (1986)  USA | BPD dx: R-DIB (≥ 7) | Clinical: In | Case-control (McLean hospital) | 86  (BPD: 27, PC: 59) | 28% | 6-12 | N/R |  |  |  |  |  | ✓ |  |  |
| Gudzer et al. (1996)  Canada | BPD dx: C-DIB-R (≥ 7) | Clinical: OUT | Cross-sectional | 98  (BPD: 41, PC: 57) | BPD: 27%, PC: 14% | BPD: 10, PC: 9.6: 7-12 | N/R |  |  |  |  |  | ✓ |  | ✓ |
| Gudzer et al. (1999)  Canada | BPD dx: C-DIB-R (≥ 7) | Clinical: OUT | Case-control | 94  (BPD: 41, PC: 53) | 14% | 9.8: 7-12 | N/R |  |  |  |  |  | ✓ |  | ✓ |
| Hallquist, Hipwell & Stepp (2015)  USA | BPD sxs: IPDE-S (cont) | Community: HR | Longitudinal cohort (PGS) | 2228 (N/R) | 100% | 14-17 | 39% |  |  |  |  | ✓ |  |  |  |
| Hecht et al. (2014)  USA | BPFS: BPFS-C | Community: LI | Case-control (LI SC) | 599  (MC: 314, NMC: 285) | MC: 51%, NMC: 50.5% | 11.3 ± .94: 10-12 | MC: 11.8% NMC: 8.1% |  |  |  |  |  | ✓ |  | ✓ |
| Horesh (2008)  Israel | BPD dx: DIB-R (≥7), DSM-IV-TR criteria based dx | Clinical: OUT, Community: POP | Case-control | 59  (BPD: 20, MDD: 19, HC: 20) | MDD: 68.42%  BPD: 95%, HC: 55% | MDD: 16.3 ± 1,  BPD: 16.4 ± 1.7,  HC: 17.5 ± 2.3 | N/R |  |  |  |  |  | ✓ |  |  |
| Infurna (2015) Germany | BPD dx: SCID-II (≥5) | Clinical: IN | Case-control | 71  (BPD: 44, PC: 47) | 100% | 15.6 ± 1.4 | N/R |  |  |  |  |  | ✓ |  |  |
| James, Berelowitz & Vereker (1996)  UK | BPD dx: R-DIB (≥7) | Clinical: IN | Case-control | 48  (BPD: 24, PC: 24) | 83.3% | BPD: 14.9 ± 1.1,  PC: 14.8 ± 1.2 | N/R |  |  |  |  |  | ✓ |  |  |
| Ludolph et al. (1990)  USA | BPD dx, PD dx: R-DIB (≥7) | Clinical: IN | Longitudinal cohort (Westen 1990) | 50  (BPD: 27, PC: 23) | 100% | BPD: 15.8, PC: 15.5: 14-18 | N/R |  |  |  |  |  | ✓ |  |  |
| Nickell et al. (2002)  USA | BPF: PAI-BOR (<37 and >38), SIDP-IV, DIB-R | Community: UND | Cross-sectional | 393  (N/R) | 54.30% | 18 | 84.9% | ✓ |  |  |  |  |  |  |  |
| Rogosch & Cichetti (2005)  USA | BPD precursors: BPD precursors composite (>1 SD above M) | Community: LI | Case-control (LI SC) | 360  (MC: 185, NMC: 175) | 48.6% | 6-12 | 82.8% "minority race/ethnicity" |  |  |  |  |  | ✓ |  | ✓ |
| Trull (2000)  USA | BPF: PAI-BOR (>38), MMPI-BPD, SIDP-IV, DIB-R | Community: UND | Cross-sectional | 421  (BF+: 197, BF-: 199) | BF+: 60.41%; BF-: 84.03% | 18 | 84.1% | ✓ |  |  |  |  |  |  |  |
| Venta et al. (2012)  USA | BPD dx, BPF: CI DSM-IV BPD (≥5), BPFSC | Clinical: IN | Cross-sectional | 106  (BPD: 14, PC: 92) | 65.1% | 14.6 ± 1.4: 12-17 | 25.5% |  |  |  |  |  | ✓ |  |  |
| Whalen et al. (2015)  USA | BPD sxs: BPFSC | Clinical: IN | Cross-sectional | 109  (MD: 38, ODD: 19, MDD: 14, ADHD: 14, BP: 10,  DD: 6, AdjD: 5, ICD: 2,  IED: 1, ASD: 1,  CD: 1, DysD: 1, PTSD: 1, SZ: 1) | 46.7% | 14.3 | 34.9% |  |  |  |  |  | ✓ |  |  |
| Wood, Parmelee & Arents (1992)  USA | BPD dx, BPD sxs: DIB-R (>7), DSM-III criteria-based dx | Clinical: IN | Case-control | 54  (BPD: 24, PC: 30) | 18.5% | 9.27: 6-12 | 61% |  |  |  |  |  | ✓ |  |  |
| Zelkowitz et al. (2001)  Canada | BPD dx: C-DIB-R | Clinical: OUT | Cross-sectional | 86  (BPD: 35, PC: 51) | BPD: 20%, PC: 7.8% | 9.8: 7-12 | N/R |  |  |  |  |  |  |  | ✓ |
| Total (*n* =28) | | | | 9686 (BPD: 919) | 63.1% | 13.8 ± 1.4 (9.4-14) | 51.5% |  | | | | | | | |
