# Supplementary material for: Parenting and personality disorder: An overview and meta-synthesis of systematic reviews
Source: PLoS One. 2019 Oct 1;14(10):e0223038. doi: 10.1371/journal.pone.0223038 (PMC6772038; doi:10.1371/journal.pone.0223038)
Supplement: S4 Table — (DOCX) [file pone.0223038.s005.docx]

| Primary studies included in systematic reviews | | | | | | | | | | | | Systematic reviews | | | | | | | |
| --- | --- | --- | --- | --- | --- | --- | --- | --- | --- | --- | --- | --- | --- | --- | --- | --- | --- | --- | --- |
|  | | | | Parent participants | | | | Offspring participants | | | |  | | | | | | | |
| Author (date) Country | PD construct: assessment (cut-point) | Setting | Design | N (clinical group/s: n) | Gender  % female | Age  M ± SD: range  years | Race  % Caucasian | N (clinical group/s: n) | Gender  % female | Age  M ± SD: range | Race  % Caucasian | Keinanen et al. (2012) | Laulik et al. (2013) | Petfield  et al. (2015) | Eyden et al. (2016) | Stepp et al. (2016) | Winsper et al. (2016) | Boucher et al. (2017) | Ibrahim  et al. (2018) |
| Abela et al. (2005)  Canada | MDD + BPD dx: SCID-II (≥5) | Community: HR | Case-control | 102 (MDD: 88, MDD + BPD: 14) | 86.3% | 40.3 ± 6.4: 27-43 | 84.3% | 140 (MDD: 120, MDD + BPD: 20) | 50.7% | 9.8 ± 2.3: 6-14 years | N/R |  |  | ✓ | ✓ |  |  |  |  |
| Barnow et al (2006)  Germany | BPD dx, CC dx: SCID-II (≥4) | Community: POP | Longitudinal cohort (SHIP) | 196 (BPD: 16, DEP: 36, CC: 28, HC: 116) | 100% | BPD: 40 ± 5.1,  DEP: 40 ± 4.2,  CC: 39.8 ± 4.5,  HC: 39.6 ± 4.5 | N/R | 257 (BPD: 23, DEP: 47, CC: 31, HC: 156) | BPD: 69.6%, DEP: 57.4%, CC: 61.3%, HC: 53.8%, | BPD: 15.5 ± 2.2, DEP: 15.5 ± 2.2, CC: 15.3 ± 2.2, HC: 15.1 ± 2.4: 11–18  years | N/R |  |  | ✓ | ✓ |  |  |  |  |
| Barnow et al (2013) Germany | BPD sxs: SCID-II (SR) | Community: POP | Longitudinal cohort (GFS) | 247  (N/R) | 100% | 44.5 ± 4.8 | N/R | 323  (N/R) | 55.1% | 19.6 ± 2.4 years | N/R |  |  |  | ✓ | ✓ |  |  |  |
| Bertino et al (2012)  Australia | PD dx: MCMI-III | Clinical + Sub-Clinical: OUT | Cross-sectional | 59  (N/R) | 79.7% | N/R | N/R | 59  (N/R) | Child: 43.3%, Adolescent: 34.5% | Child: 6.4 ± 2.5, Adolescent: 15.4 ± 1.6: 4-18 years | N/R |  |  |  | ✓ |  |  |  |  |
| Blankley et al (2015)  Australia | BPD dx: DSM-IV-R criteria-based dx | Clinical: IN + OUT, Community: POP | Case-control | 14355 (BPD: 42, HC: 14313) | 100% | 27.4 ± 6.2: 15-43 | N/R | 14355 (BPD: 42, NCC: 14313) | N/R | "newborn infants" | N/R |  |  |  | ✓ |  |  |  |  |
| Cheng (2010)  China | PD dx: PDQ-4 (>40 index; <20 control), IPDE | Community: POP | Case-control | 2786 ((PD: 181 (167 reported in results), HC: 2605)) | PD: 50%, HC: 50% | majority of sample 41-50 | N/R | 2786  ((PD: 181 (167 reported in results), HC: 2605)) | PD: 49.7%, HC: 57.2% | PD: 18-25  years | N/R |  |  |  | ✓ |  |  |  |  |
| Conroy et al. (2010)  UK | PD dx, PD+DEP dx: SCID-II | Clinical: OUT | Longitudinal Cohort | 200  (PD: 41, DEP: 39, PD+DEP: 67, HC: 53) | 100% | 30.7 ± 6.6: 16-44 | 54% | 200  (N/R) | 52% | 2 months | N/R |  | ✓ |  |  |  |  |  |  |
| Crandell, Patrick & Hobson (2003)  UK | BPD dx: SCID–NP, SCID-II (≥5), DSM-III-R criteria-based dx | Community: POP | Case-control | 20  (BPD: 8, HC: 12) | 100% | N/R | BPD: 62.5% HC: 91.7% | 20  (BPD: 8, HC: 12) | BPD: 25%,  HC: 41.7% | BPD: 65 ± 10; HC: 66 ± 7: 56-70 days | N/R |  | ✓ | ✓ | ✓ |  |  |  |  |
| Crittenden & Newman (2010)  Australia | BPD dx: DIB (>8) | Clinical: OUT, Community: POP | Case-control | 32  (BPD: 15, HC: 17) | 100% | N/R | N/R | 32  (BPD: 15, HC: 17) | N/R | 3-36 months | N/R |  |  | ✓ | ✓ |  |  |  |  |
| Delavenne et al (2008)  France | BPD dx: SIDP-IV | Clinical: OUT, Community: POP | Case-control | 34  (BPD: 17, HC: 17) | 100% | 25-38 | N/R | 34  (BPD: 17, HC: 17) | 55.9% | 3 months | N/R |  |  | ✓ | ✓ |  |  |  |  |
| DeMulder et al. (1995)  USA | PD dx: PDE | Clinical: OUT, Community: POP | Longitudinal cohort (Radke-Yarrow et al. 1992) | 89  (N/R) | 100% | 32.7 ± 4.3: 23-45 | 85% | 89  (N/R) | N/R | T3: 9.3 ± 1.1  years | N/R |  | ✓ |  |  |  |  |  |  |
| Elliot et al (2014)  Australia | BPD dx: ZAN-BPD (≥8) | Clinical: OUT, Community: POP | Case-control | 26  (BPD: 13, HC: 13) | 100% | BPD: 24.2 ± 6.7,  HC: 28.3 ± 3.5; 17-40 | N/R | 26  (BPD: 13, HC: 13) | BPD: 53.9%, HC: 38.5% | BPD: 7.7 ± 4, HC: 6.5 ± 2.7: 3-14 months | N/R |  |  | ✓ | ✓ |  |  |  |  |
| Feldman et al (1995)  Canada | BPD dx, OPD dx: DIB-R (current cut point of 6 or past score of 8) | Clinical: IN + OUT | Longitudinal cohort (Paris et al., 1994a) | 23  (BPD: 9, OPD: 14) | 100% | BPD: 38 ± 6,  OPD: 37 ± 5 | N/R | 44  (BPD: 21, OPD: 23) | BPD: 47.6%, OPD: 52.2% | BPD: 12 ± 4,  OPD: 9.5 ± 4  years | N/R |  |  | ✓ | ✓ |  |  |  |  |
| Frankel-Waldheter et al (2015)  USA | BPD dx, BPDF: SCID-II (≥5), PAI-BOR | Clinical: OUT, Community: POP | Cross-sectional | 56  (BPD: 28, HC: 28) | 100% | N/R | N/R | 56  (BPD: 28, HC: 28) | 50% | 15.4 ± 1.2 years | N/R  (7% “minority ethnic”, 4% Hispanic) |  |  |  | ✓ |  |  |  |  |
| Gratz et al (2014)  USA | BPD-H, BPD-L: BEST (>30) | Community: POP | Longitudinal cohort | 101 (BPD-H: 23, BPD-L: 78) | 100% | BPD-H: 29 ± 6.4, BPD-L: 28.4 ± 4.9; 18-42 | BPD-H: 26%,  BPD-L: 49% | 101 (BPD-H: 23, BPD-L: 78) | BPD-H: 47.8%, BPD-L: 56.4% | BPD-H: 17.4 ± 3.8,  BPD-L: 16.2 ± 3.5 months | N/R |  |  | ✓ | ✓ |  |  |  |  |
| Gunderson & Lyoo (1997)  USA | BPD dx: DIPD | Clinical: IN | Case-control | 40  (N/R) | 52.2% | N/R | N/R | 21  (BPD: 21) | 100% | 27.3 ± 4.7: 20-35 years | N/R | ✓ |  |  |  |  |  | ✓ |  |
| Guttman & Laporte (2000)  Canada | BPD dx: DIB-R | Clinical: OUT, Community: POP | Case-control | 81 Families (BPD: 26, AN: 28, HC: 27) | N/R | N/R | N/R | 81 ((BPD: 26 (27 in abstract), AN: 28, HC: 27)) | 100% | BPD: 32, AN: 22, HC: 21; 16-40 years | N/R |  |  |  |  |  |  | ✓ |  |
| Guttman & Laporte (2002)  Canda | BPD dx: DSM-III-R criteria-based dx, DIB-R (≥ 8) | Clinical: OUT, Community: POP | Case-control | 69 Families (BPD: 21, AN: 23, HC: 25) | N/R | BPD: 58 ± 11.2,  AN: 51 ± 11.2,  HC; 50 ± 9.2 | N/R | 69  (BPD: 21, AN: 23, HC: 25) | 100% | BPD: 32 ± 6.2, AN: 22 ± 5.5, HC: 21 ± 4.9; 16-40 years | N/R |  |  |  |  |  |  | ✓ |  |
| Hammen et al. (2015)  USA (Australian sample) | BPD sxs: SCID-II (≥5), IPDE, DIB-R (≥8) | Community: HR | Longitudinal cohort (MUSP) | 385  (N/R) | 100% | N/R | N/R | 385  (N/R) | 61% | 22-25 years | 92.5% |  |  |  |  | ✓ |  |  |  |
| Hans et al. (1999)  USA | PD dx: SADS-L, CAPPS | Clinical: IN + OUT | Longitudinal cohort (Jeremy & Hans, 1985) | 69  (OD: 32, NDD: 37) | 100% | OP: 27.7 ± 3.9,  NDD: 25.7 ± 3.9 | 0%  (100% African-American) | 69  (OD: 32, NDD: 37) | N/R | 10: 8.9-11.8 years | 0%  (100% African-American) |  | ✓ |  |  |  |  |  |  |
| Harvey, Stoeseel, & Herbert (2011)  USA | PD dx: MCMI-III (≥75) | Community: HR | Longitudinal cohort | 308  (N/R) | 59.09% | Mothers: 31.5 ± 6.9, Fathers: 36.5 ± 7.4 | Mothers: 62.6% Fathers: 67.5% | 184 (BPD: 184) | N/R | 36-50 months | N/R |  |  |  | ✓ |  |  |  |  |
| Herr et al (2008)  Australia | BPD sxs: SCID-q (≥5) | Community: POP | Longitudinal cohort (MUSP) | 815 (MDD: 189, DD: 83, MDD+DD: 82, HC: 461) | 100% | N/R | 92% | 815 (MDD/DD: 110) | 49.3% | 15 years | 92% |  |  | ✓ | ✓ |  |  |  |  |
| Hobson et al (2005)  UK | BPD dx: SCID-NP, SCID-II (≥5) | Clinical: OUT, Community: POP | Case-control | 32  (BPD: 10, HC: 22) | 100% | BPD: 32 ± 7, HC: 33 ± 4; 18-42 | BPD: 60%,  HC: 73% | 32  (BPD: 10, HC: 22) | BPD: 50%,  HC: 50% | BPD: 53 ± 2.8, HC: 55 ± 1.8; 47-58 weeks | N/R |  | ✓ | ✓ | ✓ |  |  |  |  |
| Hobson et al (2009)  UK | BPD dx: SCID-NP, SCID-II (≥5) | Clinical: OUT, Community: POP | Case-control | 59  (BPD: 13, DEP: 15, HC: 31) | 100% | BPD: 31.5 ± 6.5,  DEP: 28 ± 6.8, HC: 31 ± 4.5 | BPD: 69.2%  DEP: 73%  HC: 77.4% | 59  (BPD: 13, DEP: 15, HC: 31) | BPD: 46.2%,  DEP: 53.3%, NC: 41.9% | BPD: 58.5 ± 2.7, DEP: 80 ± 4, NC: 62 ± 1.9; 12-18 months | N/R |  | ✓ | ✓ | ✓ |  |  |  |  |
| Howard (1995)  USA | PD sxs: MCMI | Community: HR | Longitudinal cohort | 51  (PSA: 51) | 100% | 28.6 ± 4.9: 19-45 | 16% | 51 (N/R) | 50% | "infants" | N/R |  |  |  | ✓ |  |  |  |  |
| Jellinek et al (1991)  USA | PD dx: DSM-III-R dx criteria based dx (≥4) | Clinical: OUT | Cross-sectional | 74  (N/R) | "for 78 of the children, the patient was the mother” | N/R | N/R | 100  (PD: 17, AD: 50, MD: 28, AD + MD parent: 16) | 38% | 9.8 ± 1.9: 6-12 years | 90% "non-minority" |  |  |  | ✓ |  |  |  |  |
| Johnson et al (1999a)  USA | PD sxs: DISC-I, PDQ, DSM-IV criteria based dx | Community: POP | Longitudinal cohort (CIC) | 639  (N/R) | 100% | N/R | N/R | 639  (N/R) | 47.7% | T2: 22.3 ± 2.6: 18-28  years | 90% |  |  |  |  | ✓ |  |  |  |
| Johnson et al (1999b)  USA | BPD sxs, BPD dx: CIC-SR | Community: POP | Longitudinal cohort (CIC) | 738  (N/R) | 100% | N/R | N/R | 739  (N/R) | 48% | T2: 22 years | 90% | ✓ |  |  |  |  |  |  |  |
| Johnson et al. (2000)  USA | PD sxs, PD dx: CIC-SR, DISC-I, PDQ | Community: POP | Longitudinal cohort (CIC) | 738  (N/R) | 100% | N/R | N/R | 738  (N/R) | 50.3% | T4: 22.0 ± 2.7: 18-28  years | 90% | ✓ |  |  |  | ✓ |  |  |  |
| Johnson et al. (2001)  USA | BPD sxs, BPD dx: CIC-SR | Community: POP | Longitudinal cohort (CIC) | 793 (T4: 717) (N/R) | 100% | N/R | N/R | 793 (T4: 717) (N/R) | 49% | T4: 22.1 ± 2.7  years | 91% | ✓ |  |  |  | ✓ |  |  |  |
| Johnson et al. (2006)  USA | PD dx: PDQ, SCID-II | Community: POP | Longitudinal cohort (CIC) | 593  (N/R) | 100% | N/R | N/R | 593  (N/R) | N/R | T5: 33.1 ± 2.9  years | N/R | ✓ | ✓ |  |  | ✓ |  |  |  |
| Johnson et al (2008)  USA | PD dx: DISC-I, PDQ, SCID-II, SCID-IV NP | Community: POP | Longitudinal cohort (CIC) | 377  (N/R) | 59.4% | 33.5 ± 2.7 | 91% | 377  (N/R) | N/R | 8 years | N/R |  | ✓ |  |  |  |  |  |  |
| Kiel et al (2011)  USA | BPD-H, BPD-L: BEST (>30) | Community: POP | Cross-sectional | 99  (BPD-H: 22, BPD-L: 77) | 100% | BPD-H: 28.6 ± 6.2,  BPD-L: 28.3 ± 4.9: 18-42 | BPD-H: 22%,  BPD-L: 49% | 99  (BPD-H: 22, BPD-L: 77) | BPD-H: 45.5%, BPD-L: 58.4% | BPD-H: 17.2 ± 3.8,  BPD-L: 16.3 ± 3.7: 12-23 months | N/R |  |  | ✓ | ✓ |  |  |  |  |
| Liotti & Pasquini (2000)  Italy | BPD dx: SCID-II | Clinical: IN + OUT | Case-control | 212 (BPD: 66, PC: 146) | 100% | N/R | N/R | 212 (BPD: 66, PC: 146) | 70.75% | Majority (60.6%) <29 years | N/R | ✓ |  |  |  |  |  |  |  |
| Lyons-Ruth et al. (2013)  USA | BPD sxs: SCID-II | Community: LI | Longitudinal cohort | 56  (N/R) | N/R | N/R | 73% | 56  (N/R) | 41% | 19.7: 18-23 years | 73% |  |  |  |  | ✓ |  |  |  |
| Macfie and Swan (2009)  USA | BPD dx: SCID-II (≥5), PAI (cont) | Community: HR | Case-control | 60  (BPD: 30, PC: 30) | 100% | N/R | N/R | 60  (BPD: 30, PC: 30) | 50% | 5.3 ± 0.9: 4-7 years | 88% |  |  | ✓ | ✓ |  |  |  |  |
| Macfie et al (2014)  USA | BPD dx, BPDF: SCID-II (≥5), PAI (cont) | Clinical: OUT, Community: HR | Case-control | 62  (BPD: 31, HC: 31) | 100% | BPD: 31.7 ± 5,  HC: 32.8 ± 5.4 | N/R | 62  (BPD: 31, HC: 31) | 53.2% | 4-7 years | 92% |  |  |  | ✓ |  |  |  |  |
| Marantz & Coates (1991)  USA | BPD dx: DIB (cont) | Clinical: OUT | Case-control | 33  (N/R) | 100% | 32 | 50% | 33  (GID: 16, NoGID: 17) | 0% | 8 years | 50% |  |  |  | ✓ |  |  |  |  |
| Newman et al (2007)  Australia | BPD dx: DSM-IV, DIB-R (≥8) | Clinical: OUT, Community: POP | Case-control | 34  (BPD: 14, HC: 20) | 100% | BPD: 28.6 ± 6.9,  HC: 33.4 ± 4.4 | N/R | 34  (BPD: 14, HC: 20) | BPD: 57.1%; HC: 55% | BPD: 15.6 ± 7.8,  HC: 16.1 ± 6.3: 3-36 months | N/R |  | ✓ | ✓ | ✓ |  |  |  |  |
| Reinelt et al (2014)  Germany | BPD sxs: SCID-II (≥5) | Community: POP | Longitudinal cohort (GFS) | 230  (N/R) | 100% | T0: 39.9 ± 4.5 | N/R | 295  (N/R) | 54.9% | T1: 19.4 ± 2.2 | N/R |  |  |  | ✓ | ✓ |  |  |  |
| Schact et al (2013)  UK | BPD dx: SCID-II (≥5) | Community: HR | Longitudinal cohort (Conroy, 2010) | 39  (BPD: 20, HC: 19) | 100% | BPD: 34 ± 7,  HC: 36.2 ± 6.4; 22-46 | BPD: 60%,  HC: 79% | 39  (BPD: 20, HC: 19) | BPD: 60%,  HC: 42.11% | BPD: 48.6 ± 5.6,  HC: 53.1 ± 9.9  months | N/R |  |  | ✓ | ✓ |  |  |  |  |
| Schuppert (2012)  Netherlands | BPD dx, PD dx, BPF: SCID-II (≥2), PDQ-4 | Clinical: “Referred to emotion regulation training” | Case-control | 145  (N/R) | 100% | N/R | N/R | 145 (BPD: 96-101, HC: 44) | 92% | 14-19 years | N/R |  |  |  |  |  | ✓ |  |  |
| Schuppert (2014)  Netherlands | BPT, BPD sxs, PDT: BPDSI-IV-ado, SCID-II, PDQ-4+ | Community: POP | Cross-sectional | 101  (N/R) | 100% | N/R | 83.4% | 101  (N/R) | 96% | 16.3 ± 1.15: 14-18.7 years | N/R |  |  |  |  |  | ✓ |  |  |
| Stepp et al. (2013)  USA | BPD sxs: IPDE, SCID-NP, SCID-II | Community: HR | Longitudinal cohort (OADP) | 1392 (N/R) | 50.4% | N/R | N/R | 816  (N/R) | 58.8% | 17.2 ± 1.3 years | N/R |  |  |  | ✓ | ✓ |  |  |  |
| Stepp et al. (2014)  USA | BPD sxs: IPDE-BOR | Community: HR | Longitudinal cohort (PGS) | 2212 (N/R) | N/R | 37.8 ± 8.6: 21-83 | N/R | 2212 (N/R) | 100% | 14-17 years | 41.2% |  |  |  |  | ✓ | ✓ |  |  |
| Stepp et al. (2015)  USA | BPD sxs IPDE-B | Community: HR | Longitudinal cohort (PGS) | 113  (N/R) | 100% | N/R | N/R | 113  (N/R) | 100% | 16-18 years | 33% |  |  |  |  | ✓ |  |  |  |
| Weiss et al (1996)  Canada | BPD dx, OPD dx: DIB-R (≥8), CDIB | Clinical: IN | Case-control | 23  (BPD: 9, OPD: 14) | 100% | N/R | N/R | 44  (BPD: 21, OPD: 23) | N/R | BPD: 12, OPD: 9.5 years | N/R |  |  | ✓ | ✓ |  |  |  |  |
| Whalen et al. (2015)  USA | BPD-H, BPD-L: BEST | Community: POP | Cross-sectional | 101  (BPD: 23) | 100% | 28.6 ± 5.3: 18-42 | 44% | 101 (BPD: 23) | 54.5% | 16.5 ± 3.6; 12-23 months | N/R |  |  |  | ✓ |  |  |  |  |
| White et al (2001)  USA | BPD dx, BPD + MDD dx: SCID-II (>5), IPDE, DIB-R (>8) | Clinical: OUT | Case-control | 87  (BPD: 17, HC: 25, MDD: 25, BPD + MDD: 20) | 100% | BPD: 30 ± 3.1,  MDD: 29.9 ± 3,  BPD + MDD: 29 ± 4,  HC: 18.9 ± 6 | BPD: 52%, MDD: 60%, BPD + MDD: 55%,  NC: 60% | 87  (BPD: 17, MDD: 25,  BPD + MDD: 20,  HC: 25) | BPD: 47.1%; MDD: 44%; BPD+MDD: 40%;  HC: 48% | BPD: 98 ± 5,  MDD: 99 ± 4.1, BPD + MDD: 101 ± 4.4, NC: 100 ± 4.6 months | N/R |  |  | ✓ | ✓ |  |  |  |  |
| Wilson & Durbin (2012)  USA | PD sxs: IPDE-S (cont) | Community: POP | Cross-sectional | 145  (N/R) | N/R | Mothers: 37 ± 5,  Fathers: 39 ± 6: 23-57 | Mothers: 73%, Fathers: 74% | 145  (N/R) | 47% | 4.5 ± .9: 3-6 years | N/R |  |  |  | ✓ |  |  |  |  |
| Winsper, Zanarini & Wolke (2012)  UK | BPD dx, BPD sxs: UK-CI-BPD | Community: POP | Longitudinal cohort (ALSPAC) | 6050 (N/R) | 100% | N/R | N/R | 6050 (N/R) | 45.9% | 11.7: 10.4-13.6 years | 48.2% |  |  |  |  | ✓ | ✓ |  | ✓ |
| Winsper, Wolke & Lereya (2015)  USA | BPD sxs: UK-CI-BPD | Community: POP | Longitudinal cohort (ALSPAC) | 6050 (N/R) | 100% | N/R | N/R | 6050 (N/R) | 51.40% | 11-12 years | N/R |  |  |  |  | ✓ |  |  |  |
| Wolke et al. (2012)  UK | BPD sxs: UK-CI-BPD | Community: POP | Longitudinal cohort (ALSPAC) | 6050 (N/R) | 100% | N/R | N/R | 6050 (N/R) | 46% | 11.8 years | 48% |  |  |  |  | ✓ |  |  |  |
| Zalewksi et al. (2014)  USA | BPD sxs: IPDE-BOR | Community: HR | Longitudinal cohort (PGS) | 1598 (BPD: 128) | 100% | N/R | N/R | 1598 (N/R) | 100% | 15-17 years | 41% |  |  |  | ✓ |  |  |  |  |
| Total (*n =* 54) | | | | 49079 (BPD: 790) | 94.5% | 34.2 ± 5.4 (22.8-47.9) | 61.15% | Infants: 14,686  Child: 1,801  Adolescent: 27,856  Adult: 4,620 | Infants: 52.6%  Child: 41.9%  Adolescent: 69.2%  Adult: 66.8% | Infants: 10 ± N/R (10-33) months  Child: 6.9 ± 1.4 (2.8-5.8) years  Adolescent: 14.7 ± 1.6 (13-16.9) years  Adult: 23.5 ± 3 (18.3-31.3) years | Infants: N/R  Child: 80%  Adolescent: 50%  Adult: 87.8% |  | | | | | | | |
