# Supplementary material for: Parenting and personality disorder: An overview and meta-synthesis of systematic reviews
Source: PLoS One. 2019 Oct 1;14(10):e0223038. doi: 10.1371/journal.pone.0223038 (PMC6772038; doi:10.1371/journal.pone.0223038)
Supplement: S1 Text — (DOCX) [file pone.0223038.s006.docx]

AA = alcohol abuse

Ab-DIB = abbreviated diagnostic interview of borderlines

AD = anxiety disorder

ADHD = attentive deficit hyperactivity disorder

ADjD = adjustment disorder

AGOR = agoraphobia

ALSPAC = Avon longitudinal study of population and community

AN = anorexia nervosa

ANX = anxiety

AP = abusive parents

ASD = acute stress disorder

ASPD = antisocial personality disorder

AUD = alcohol abuse and/or alcohol dependence diagnosis

AVPD = avoidant personality disorder

Axis I = axis I disorder

Axis II = axis II disorder

BD = bipolar disorder

BEST = borderline evaluation of severity over time

BF- = borderline features not present

BF+ = borderline features present

BI = bipolar-I disorder

BPD = borderline personality disorder

BPD-Active = active BPD

BPD-H = high BPD

BPD-L = low BPD

BPD-Life = lifetime BPD (met criteria in past but not in present)

BPDSI-IV-ado = borderline personality disorder severity index for DSM IV - adolescent version

BPF = borderline personality features

BPFS = borderline personality features scale

BPFS-C = borderline personality features scale - children

BP-II = bipolar II disorder

BPRC = borderline personality related characteristics

BPS = borderline personality symptoms

BPT = borderline personality traits

BSI = borderline severity index

BUL = bulimia

CA = cluster A personality disorder

CAC = cluster A or C personality disorder

CAPPS = current and past psychopathology scale

CB = cluster B personality disorder

CC = cluster C personality disorder

CD = conduct disorder

C-DIB =child diagnostic interview for borderline

C-DIB-R = child diagnostic interview for borderline revised

CEAS = childhood experiences adulthood stress study

CI = child interview

CIC = children in the community study

CIC-SR = children in the community study – self report

CLPS = the collaborative longitudinal personality disorders study

CPNI = Coolidge personality and neuropsychological inventory for children

DD = depressive disorder

DEP = depression

DIB = diagnostic interview for borderlines

DIB-2 = diagnostic interview for borderlines - 2nd edition

DIB-R = diagnostic interview for borderlines - revised

DIPD = diagnostic interview for personality disorders

DIPD-IV = diagnostic interview for DSM-IV personality disorders

DIPD-R = diagnostic interview for DSM-III-R personality disorders

DISC-I = diagnostic interview schedule for children

D-OPD = dysthymic OPD

DSM = diagnostic and statistical manual of mental disorders

Dx = diagnosis

DysD = dysthymic disorder

E-Risk = environmental risk study

EXT/INT = externalising/internalising disorder

FOR = forensic

FPI = forensic prisoner inpatient

GFS = Greifswald family study (subpopulation of SHIP)

GID = gender identity disorder

HC = healthy comparisons/controls

HP = hospital personnel

HPD = histrionic personality disorder

HR = high risk

HSNS = hypersensitivity narcissism scale

Hx = history

ICD = impulse control disorder

ICD-10 = international classification of diseases – 10th edition

IED = intermittent explosive disorder

IN = inpatients

INC = incarcerated

IPDE = international personality disorder examination

IPDE-BOR = international personality disorder examination – borderline

IPDE-S = international personality disorder examination screener

IS = intensive service residential or day treatment (no BPD)

LBSI = lifetime borderline symptom index

LI = low income

LI SC = low income summer camp

MBPD = Minnesota borderline personality disorder scale

MC = maltreated children

MCMI III = Millon clinical multiaxial inventory – III

MD = mood disorder

MDD = major depressive disorder

MDE = major depressive episode

MMPI-BPD = Minnesota multiphasic personality inventory - BPD

MP = maltreating parent

MSI-BPD = McLean’s screening instrument for BPD

MTFS = Minnesota twin family study

MUSP = Mater University of Queensland study of pregnancy

N/A = not applicable

N/R = not reported

NAP = non-abusive parents

NC = non-clinical

NDD = non-drug dependent

NEU = neurotic subgroup

NMC = non-maltreated children

NMP = non-maltreating parent

NoBPD = no borderline personality disorder

NoDEP = no depression

NoGID = no gender identity disorder

NPD = narcissistic personality disorder

NPI = narcissistic personality inventory

OADP = Oregon adolescent depression project

OCPD = obsessive compulsive personality disorder

OD = opioid dependent

ODD = oppositional defiant disorder

OPD = other personality disorder (non-borderline)

OUT = outpatients

PAARC = Pittsburgh adolescent research center

PAI-BOR = personality assessment inventory - borderline

PC = psychiatric control/comparison

P-D = panic disorder

PD = personality disorder

PDE = personality disorder examination

PDQ = personality diagnostic questionnaire

PDQ-4 = personality diagnostic questionnaire - DSM IV axis II personality disorders

PDQ-R = personality diagnostic questionnaire – revised

PDT = personality disorder traits

PGS = Pittsburgh girls study

POP = general population

PR = prisoner

PSA = poly substance abusing

PSY = psychosis

PTSD = posttraumatic stress disorder

R-DIB = revised diagnostic interview for borderlines

REF = reference group

SA = substance abuse

SADS-L = schedule for affective disorders and schizophrenia—life version

SCID-II = structured clinical interview for diagnosis of axis II disorders

SCID-NP = structured clinical interview for diagnosis – non-patient edition

SCID-PQ = structure clinical interview for diagnosis – personality questionnaire

SCID-q = structured clinical interview for diagnosis - screening questionnaire

SH = self-harm

SHIP = study of health in Pomerania, Germany

SIDP-IV = structured interview for DSM-IV personality

SIPD = Structured interview for DSM-III personality disorders

SR = self-report

ST-BPD = sub threshold BPD

STPD = schizotypal personality disorder

SUIC = suicidality

SWAP = Shelder-Westen assessment procedure

SWAP-200-A = Shelder-Westen assessment procedure 200-item q-sort for adolescents

SZ = schizophrenia

SZ-PD = schizotypal personality disorder

U-DEP = unipolar depression

UK-CI-BPD = United Kingdom child interview – BPD

UND = undergraduates

ZAN-BPD = Zanarini rating scale for BPD
